# Disruption of APOL1-miR193a Axis Induces Disorganization of Podocyte Actin Cytoskeleton

**DOI:** 10.1038/s41598-019-39376-y

**Published:** 2019-03-05

**Authors:** Vinod Kumar, Nitpriya Paliwal, Kamesh Ayasolla, Himanshu Vashistha, Alok Jha, Nirupama Chandel, Sheetal Chowdhary, Moin A. Saleem, Ashwani Malhotra, Praveen N. Chander, Karl Skorecki, Pravin C. Singhal

**Affiliations:** 1 Immunology and Inflammation Center, Feinstein Institute for Medical Research and Zucker School of Medicine at Hofstra-Northwell, New York, USA; 2 Ochsner Clinic, New Orleans, USA; 3 Academic Renal Unit, University, Bristol, UK; 4 New York Medical College, Valhalla, NY USA; 5 Technion – Israel Institute of Technology, and Rambam Health Care Campus, Haifa, Israel

## Abstract

APOL1-miR193a axis participates in the preservation of molecular phenotype of differentiated podocytes (DPDs). We examined the hypothesis that APOL1 (G0) preserves, but APOL1 risk alleles (G1 and G2) disrupt APOL1-miR193a axis in DPDs. DPDG0s displayed down-regulation of miR193a, but upregulation of nephrin expression. DPDG1s/G2s exhibited an increase in miR193a and down-regulation of the expression of adherens complex’s constituents (CD2AP, nephrin, and dendrin). DPDG0s showed decreased Cathepsin L, enhanced dynamin expressions, and the intact actin cytoskeleton. On the contrary, DPDG1s/G2s displayed an increase in Cathepsin L, but down-regulation of dynamin expressions and disorganization of the actin cytoskeleton. APOL1 silencing enhanced miR193a and Cathepsin L, but down-regulated dynamin expressions. DPDG1s/G2s displayed nuclear import of dendrin, indicating an occurrence of destabilization of adherens complexes in APOL1 risk milieu. These findings suggest that DPDG1s and DPDG2s developed disorganized actin cytoskeleton as a consequence of disrupted APOL1-miR193a axis. Interestingly, docking and co-labeling studies suggested an interaction between APOL1 and CD2AP. APOL1^*G1*/*G1*^ and APOL1^*G1*/*G2*^ transgenic mice displayed nuclear import of dendrin indicating destabilization of adherens complexes in podocytes; moreover, these mice showed a four-fold increase in urinary albumin to creatinine ratio and development of focal segmental glomerular lesions.

## Introduction

African Americans carrying APOL1 renal risk alleles have been reported to develop chronic kidney disease (s) at a several-fold higher rate when compared to European Americans^1–3^. We and others have reported observations in multiple experimental platforms, which is consistent with the premise that it is a consequence of gain of injury from cytotoxic effects of overexpressing APOL1 risk alleles^4–15^. This formulation is also consistent with the apparent dispensability of APOL1 for podocyte and kidney health in many species^10,11^. On the other hand, a recessive inheritance mode for kidney disease risk in association with the APOL1 risk alleles in genetic epidemiologic studies is more consistent with a loss rather than a gain of function by APOL1 risk alleles^16–18^. Recently, we have reported that APOL1 wild-type (G0) preserves the podocyte molecular phenotype in adverse milieus associated with enhanced miR193a levels^19^. We now hypothesize that APOL1-miR193a axis preserves the integrity of the actin cytoskeleton in differentiated podocytes through stabilization of the adherens complex (AC), while disruption of APOL1-miR193a axis in podocytes expressing APOL1 risk alleles induces loss of this function.

Optimal expression of the AC proteins is considered to be an integral part of podocyte health^20,21^. Nephrin is one of the most important constituents of the ACs and is transcribed by Wilms tumor type (WT) 1 transcription factor^20,22^. Since miR193a inversely regulates the expression of WT1, it also inversely regulates the transcription of nephrin in podocytes^19,22,23^. A decrease in nephrin expression disintegrated the ACs and resulted in nuclear import of dendrin, followed by the activation of a pro-apoptotic pathway^24^. In *Danio rerio* (Zebrafish), silencing of its endogenous APOL1 contributed to altered expression of nephrin in nephrocytes as well as in the development of a dysfunctional glomerular filtration barrier^25^. These investigators suggested a role of Zebrafish APOL1 in the maintenance of the glomerular filtration barrier. However, its participation in the stability of ACs was not investigated in this model. On the other hand, an overexpression in Zebrafish pro-nephrons of exogenous human APOL1 non-risk and risk variants did not fully recapitulate a Zebrafish phenotype consistent with human APOL1 renal risk nephropathy under Puromycin Aminonucleoside (PAN) challenge^26^. We are hypothesizing that enhanced miR193a expression resulting as a consequence of mutation in the APOL1 gene destabilizes the ACs through decreased nephrin expression.

The nuclear dendrin transcribes Cathepsin (CTS) L^27^, which promotes the degradation of synaptopodin, CD2AP, and dynamin through its proteolytic activity in podocytes^27^. Optimal expression of synaptopodin and dynamin is essential for the maintenance of the integrity of the podocyte actin cytoskeleton^27^. Therefore, a decrease in any of the constituents of the ACs could destabilize the complex and induce disorganization of the actin cytoskeleton in podocytes^27^. The role of CD2AP in the maintenance of the ACs has been studied in the past^27^. CD2AP-deficient mice developed massive proteinuria and nephrotic syndrome at approximately four weeks of age and succumbed to renal failure at 6–10 weeks of age^28^. Since the kidney phenotype of CD2AP-deficient animals could be rescued with a podocyte-specific expression of CD2AP, it has been suggested that the kidney dysfunction would be a consequence of the loss of CD2AP in the podocytes^29^.

PH-dependent ion selectivity has been attributed for APOL1’s effects in both the endosomal and the plasma membranes. Endosomal acidification initiates exogenous APOL1’s insertion in the membrane contributing to anion-selective permeability; the latter is responsible for APOL1’s pleiotropic effects in intracellular compartments^30^. Although the endogenous APOL1 is also inserted in the membranes of acidified exocytic vesicles targeted to the plasma membrane, however, it inhibits anion permeability at neutral pH of the plasma membrane^14,31^. Since APOL1 is also a plasma membrane protein, it carries a potential to interact with the AC constituents.

In the present study, we have examined the role of APOL1– miR193a axis in the maintenance of stability of the ACs and the integrity of actin cytoskeleton in human differentiated podocytes. Also, we have evaluated the effects of disruption of APOL1-mIR193a axis on the stability of the ACs and the organization of actin filaments in podocytes expressing APOL1 risk alleles. We used interventions which resolved the defective signaling in adverse milieus and podocytes expressing APOL1 risk alleles.

## Results

### Evaluation whether APOL1 and Nephrin preserve the stability of adherens complexes (ACs)

**Figure 1:**
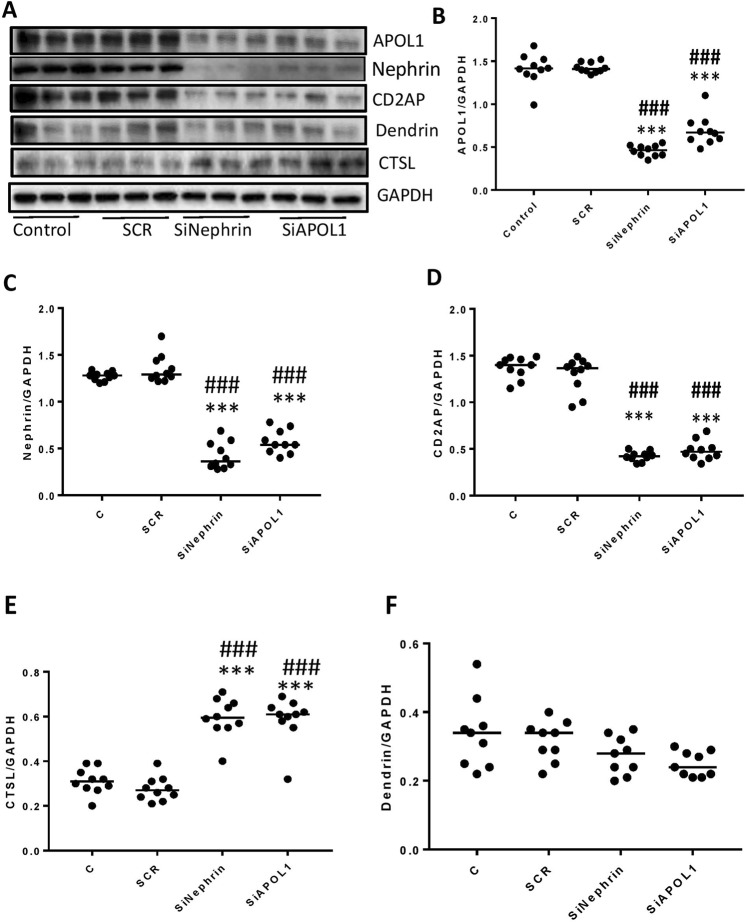
Evaluation of the effects of lack of APOL1 and Nephrin on the stability of adherens complex. (**A**) DPDS were transfected with either scrambled (SCR), APOL1- or nephrin-siRNAs (n = 10). Protein blots of control (**C**), DPD-transfected with SCR/SiRNA nephrin/siRNA APOL1 were probed for CD2AP, nephrin, APOL1, dendrin, and CTSL (n = 10). The same blots were reprobed for GAPDH. Gels from three different cellular lysates are displayed. (**B**–**F**) Cumulative densitometric data for each variable (protein/GAPDH) are shown as dot plots. ***p < 0.001 vs respective control and SCR; ^###^p < 0.001 vs. respective control and SCR. For clarity lanes were cropped from different gels. Full-length blots are presented in Fig. S1.

To evaluate whether lack of either APOL1 or nephrin in DPDs would disrupt the complex equally and result in enhanced CTSL expression, DPDS were transfected with either scrambled (SCR), APOL1- or nephrin-siRNAs (n = 10). Protein blots of control (C), DPD-transfected with SCR/siRNA nephrin/siRNA APOL1 were probed for CD2AP, nephrin, APOL1, dendrin, and CTSL. The same blots were reprobed for GAPDH. Gels from three different cellular lysates are shown in Fig. 1A. Cumulative densitometric data for each variable are shown as dot plots (Fig. 1B–F). DPDs silenced for either APOL1 or nephrin displayed attenuated expression of CD2AP (Fig. 1A and D) but enhanced expression of CTSL (Fig. 1A and E). These findings suggest that both nephrin and APOL1 are required to maintain the stability of the ACs in human DPDs.

### Evaluation of the relationship between APOL1 and AC constituents

**Figure 2:**
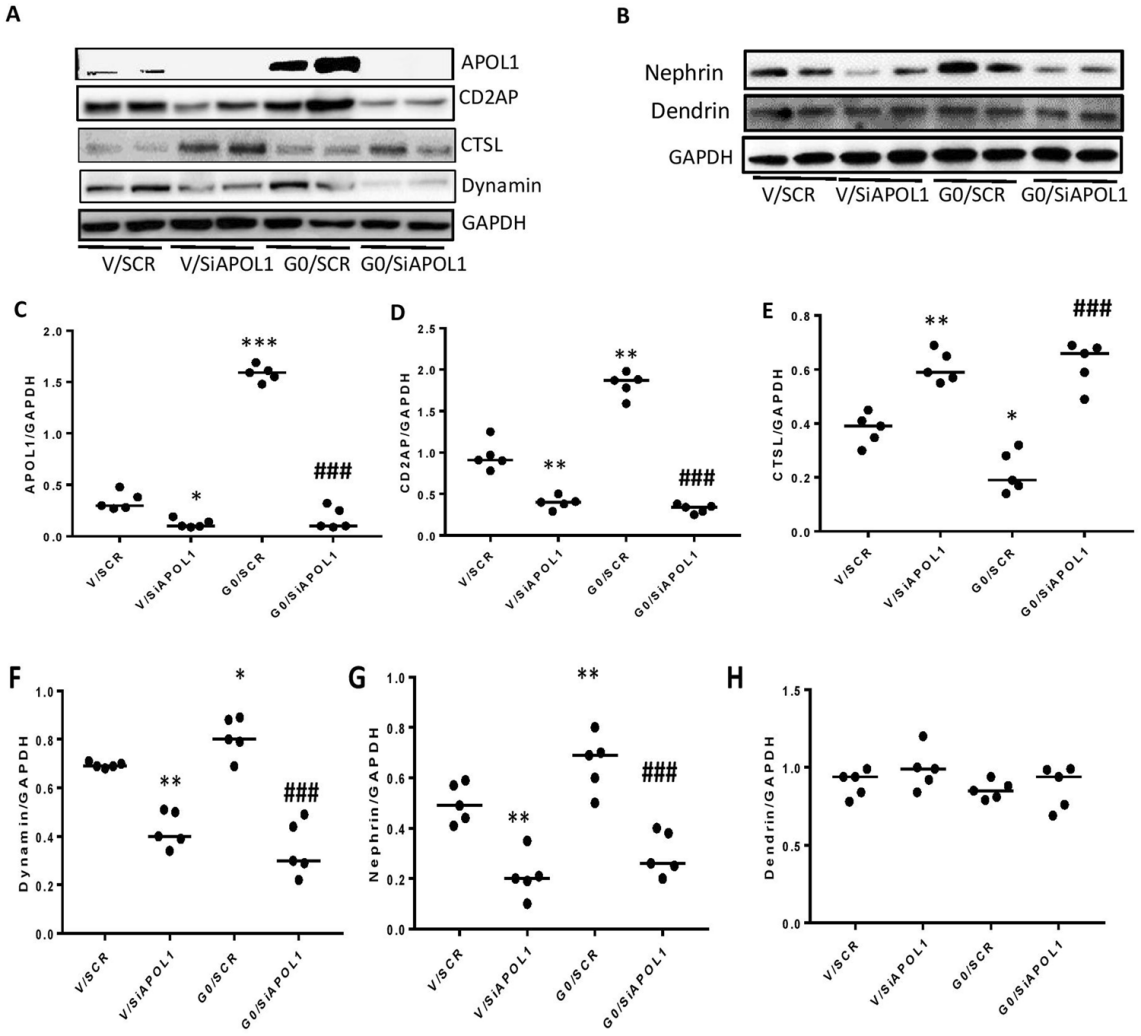
Effect of Ectopic overexpression or silencing of APOL1 on the stability of adherens complexes. (**A**) PDs stably expressing vector (PDV) and ectopic APOL1G0 (PDG0) were differentiated for ten days and the transfected with either scrambled or APOL1 siRNA (n = 5). Protein blots were probed for APOL1, CD2AP, CTSL, and dynamin and reprobed for GAPDH. The Gels from two different lysates are displayed. (**B**) Protein blots from the above lysates were probed for nephrin and dendrin and reprobed for GAPDH. The gels from two different lysates are shown. (**C**–**H**) Densitometric data for each variable (protein/GAPDH) are shown as dot plots. (**C**) *P < 0.05 compared with V/SCR; ***P < 0.001 compared with V/SCR; ^###^P < 0.001 compared with G0/SCR. (**D**) **P < 0.01 compared with V/SCR; ^###^P < 0.001 compared with G0/SCR. (**E**) *P < 0.0.5 compared with V/SCR; **P < 0.01 compared with V/SCR; ^###^P < 0.001 compared with G0/SCR. (**F**) *P < 0.05 compared with V/SCR; **P < 0.01 compared with V/SCR; ^###^P < 0.001 compared with G0/SCR. (**G**) **P < 0.0a compared with V/SCR; ^###^P < 0.001 compared with G0/SCR. For clarity lanes were cropped from different gels. Full-length blots are presented in Fig. S2.

We hypothesized that if silencing of APOL1 destabilizes the complex, then overexpression of APOL1 would enhance the stabilization of the AC further. PDs-stably expressing vector (PDV) and APOL1G0 (PDG0) were differentiated for ten days and then transfected with either scrambled or APOL1 siRNA (n = 5). Protein blots were probed for APOL1, CD2AP, CTSL, and dynamin and reprobed for GAPDH. The same lysates were reprobed for nephrin, dendrin, and GAPDH. Gels from two different lysates are displayed in Fig. 2A and B). Densitometric data for each variable are shown as dot plots (Fig. 2C–H). Silencing of APOL1 (Fig. 2A and C) decreased the expression of CD2AP (Fig. 2A and D) and dynamin (Fig. 2A and E) but increased the expression of CTSL (Fig. 2A and E). On the other hand, DPDG0 displayed enhanced expression of CD2AP (Fig. 2A and D) and minimal expression of CTSL (Fig. 2A and E). Conversely, silencing of APOL1 decreased the expression of nephrin in both Vector and APOL1G0 expressing podocytes (Fig. 2B and G). These findings indicate that lack of APOL1 downregulates the expression of AC constituents, but the presence of APOL1 increases their expressions.

### Evaluation of the relationship between APOL1 and CD2AP

**Figure 3:**
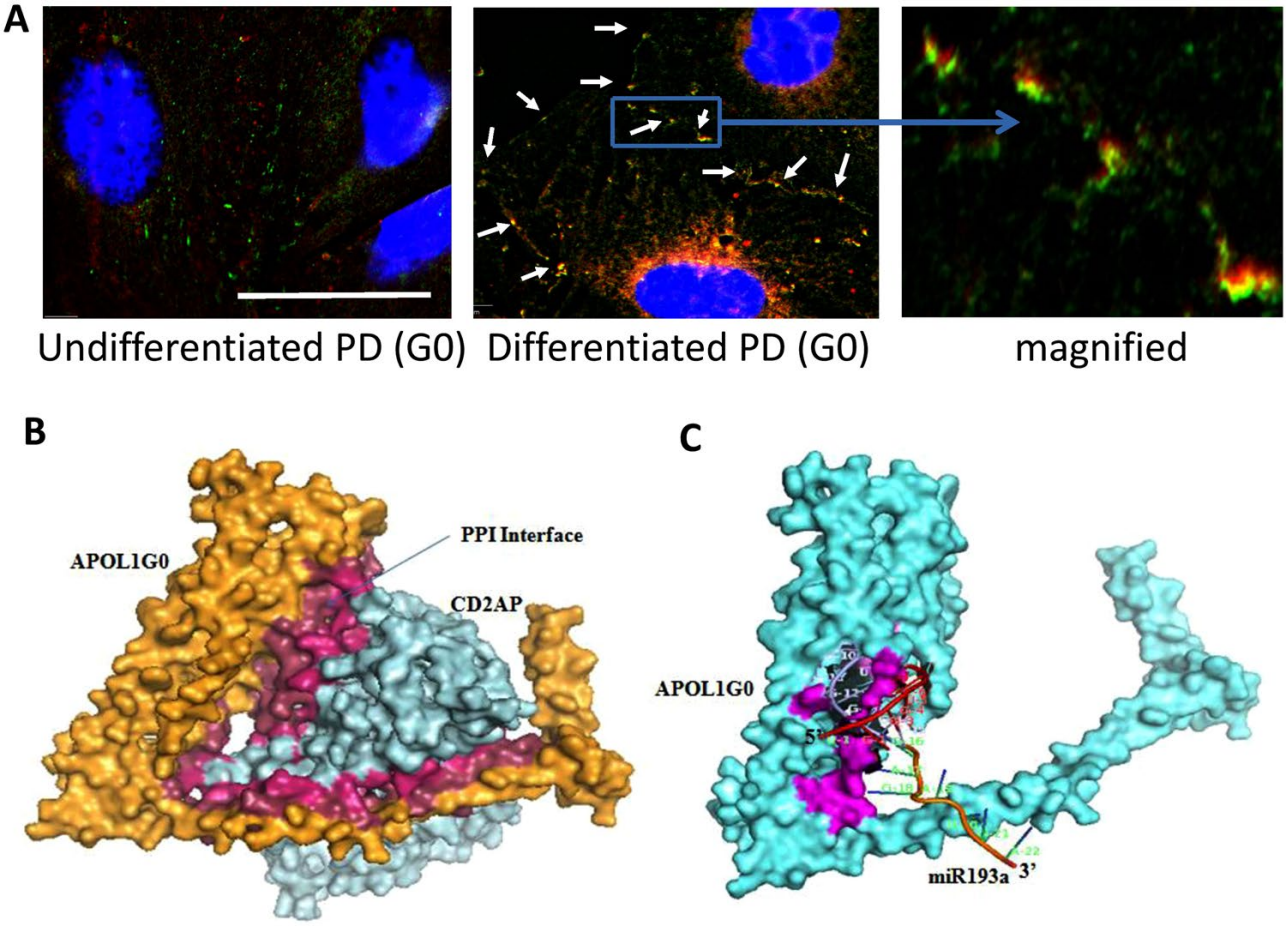
An interaction between APOL1 and CD2AP at the plasma membrane. (**A**) Undifferentiated and differentiated PDG0 grown on coverslips were co-labeled for APOL1 and CD2AP. Representative fluoromicrographs are shown. Undifferentiated PDs displayed scattered labeling for APOL1 (red fluorescence) and CD2AP (green fluorescence). Differentiated PD showed co-labeling for APOL1 and CD2AP (yellow fluorescence, indicated by white arrows) at adherent junctions. Horizontal solid white bar represents the scale as 100 µm. (**B**) The CD2AP binds to the APOL1 in the carboxy-terminal region and residues from Membrane Addressing Domain (MAD) of APOL1 are also interacting with residues of CD2AP. Hotspot residues in the protein-protein interaction interface of APOL1G0 (Raspberry) and CD2AP (Warm pink). (**C**) The interaction interface of APOL1G0 and miR193a has a total interface area of 1339.1 Å^2^ and the solvation free energy gain upon formation of interface Δ^i^G is −34.3 kcal/mol. The solvation free energy of folding for the corresponding structure ΔG is −263.1 kcal/mol. The hotspot residues (magenta) in the miRNA-Protein interaction interface of APOL1G0 and miR193a. The 5′ end of miR193a binds to the 3′ UTR region of APOL1G0 mRNA (36) and displayed symbolically on protein surface.

We next asked whether APOL1 has an interaction with any of the constituents of ACs. To determine APOL1’s link with CD2AP, undifferentiated and differentiated APOL1G0 expressing Podocytes were grown on coverslips. Podocytes were co-labeled for APOL1 and CD2AP and examined under a confocal microscope. Representative fluoromicrographs are shown in Fig. 3A. Both APOL1 and CD2AP showed partial co-labeling at adherens junctions of podocytes.

### Protein-protein Interaction (PPI) Interface analyses of APOL1G0 and CD2AP

To evaluate protein-protein interaction between APOL1 and CD2AP, we have carried out bioinformatics analysis^32^. The details are described in supplementary data (S.Fig. 3B). The CD2AP binds to the APOL1 in the carboxy-terminal region and residues from Membrane Addressing Domain (MAD) of APOL1 are also interacting with residues of CD2AP (Fig. 3B).

### APOL1G0 and miR193a interactions

Since APOL1 forms an axis with miR193a, we have carried out bioinformatics analysis to evaluate binding sites. The details are described in supplementary data (S. Fig. 3C). The residues of miR193a that correspond to the miR193a binding site at the 3′UTR region of the APOL1 mRNA (36)  are displayed symbolically on protein surface (Fig. 3C). Also, there are seven hydrogen bonds in the interface of APOL1G0 and miR193a complex.

### APOL1 risk alleles destabilize the ACs

**Figure 4:**
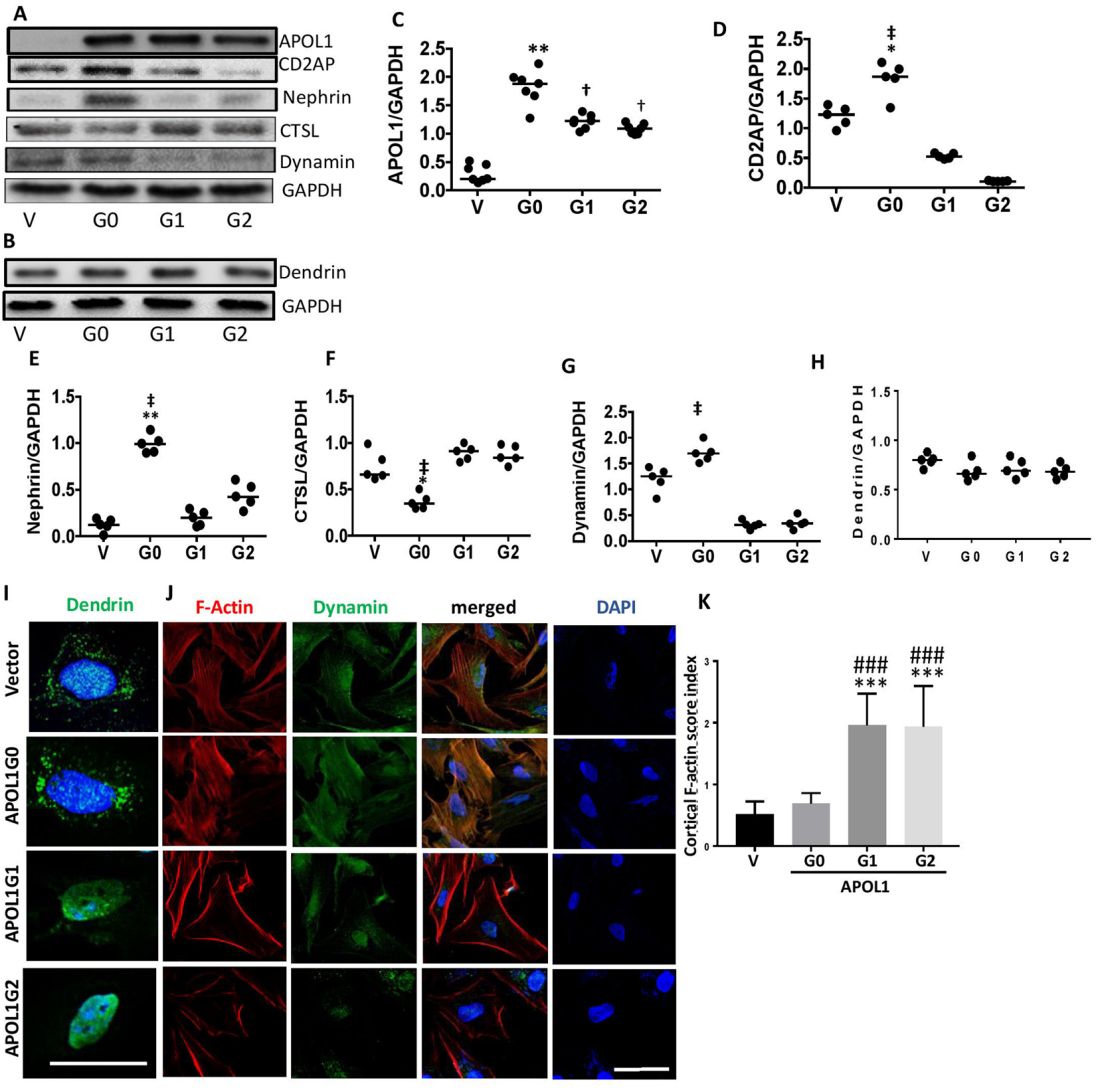
APOL1 risk alleles destabilize the adherens complex. (**A**) Protein blots of DPDs stably over-expressing Vector (V), G0, G1, and G2 were probed for APOL1, nephrin, CD2AP, dynamin, CTSL, and GAPDH (n = 5–7). Representative gels are displayed. (**B**) Protein blots of the above lysates were probed for dendrin and reprobed for GAPDH (n = 5). (**C**–**H**) Cumulative densitometric data on each variable (protein/GAPDH) are shown as dot plots. *<0.05 compared with respective V; **P < 0.01 compared with respective V; ^†^P < 0.05 compared with respective G0; ^‡^P < 0.01 compared with respective G1 and G2. (**I**) PDs expressing vector, G0, G1, and G2 grown on coverslips were differentiated and labeled for dendrin (n = 3). Nuclei were stained with DAPI. Cells were imaged under a confocal microscope. Representative fluoromicrographs are displayed. DPDs expressing vector and G0 showed predominantly cytosolic dendrin (indicated by green fluorescence). However, DPD expressing G0 and G1 showed predominantly nuclear localization of dendrin. Horizontal solid white bar represents the scale as 100 µM. (**J**) PDs expressing vector, G0, G1, and G2 grown on coverslips were differentiated (n = 3). Subsequently, cells were co-labeled for F-actin (by phalloidin) and dynamin. Nuclei were stained with DAPI. Cells were examined under a confocal microscope. Representative fluoromicrographs are shown. DPDs expressing vector and G0 displayed well- spread and intact actin filaments (indicated by red fluorescence). DPDs expressing G1 and G2 displayed predominantly cortical labeling for F-actin. DPDs expressing vector and G0 displayed moderate expression of dynamin (indicated by green fluorescence). DPDs expressing G1 and G2 displayed minimal expression of dynamin. Horizontal solid white bar represents the scale as 50 µm. (**K**) The cortical F-actin score (CFS) index was calculated under control and experimental conditions. DPDAPOLG1 (G1) and DPDAPOL1G2 (G2) displayed an increased CFS index compared with DPDV (Vector) and DPDAPOL1G0 (G0). ***P < 0.001 compared with V and G0; ^###^P < 0.001 compared to V and G0. For clarity lanes were cropped from different gels. Full-length blots are presented in Fig. S4.

To determine the role of *APOL1G0* and *APOL1* risk alleles in sustaining the stability of the adherens complex, protein blots of DPDs-stably over-expressing Vector (V), APOL1G0, APOL1G1, and APOL1G2 were probed for APOL1, nephrin, CD2AP, and dynamin. Representative gels are displayed in Fig. 4A and B. Cumulative densitometric data on each variable are shown as dot plots (Fig. 4C–H). DPDG0s displayed higher expressions of nephrin, CD2AP, and dynamin but lower expression of CTSL when compared to DPDG1s and DPDG2s (Fig. 4A–G). These findings suggest that there is destabilization of the ACs in DPDs expressing APOL1 risk alleles.

Moreover, an increase in CTSL generation by DPDs expressing APOL1G1 and APOL1G2 suggested the nuclear import of dendrin in these cells. To confirm the nuclear localization of dendrin, DPDs expressing vector, G0, G1, and G2 (grown on coverslips) were labeled for dendrin. Nuclei were stained with DAPI. Cells were imaged under a confocal microscope. Representative fluoromicrographs are shown in Fig. 4I. DPDs expressing vector and G0 displayed predominant labeling for dendrin in the cytosol, whereas, DPDs expressing G1 and G2 displayed significant nuclear localization of dendrin (Fig. 4I).

To determine the effect of APOL1 risk alleles on the DPDs’ expression of dynamin and the integrity of actin cytoskeleton, DPDs expressing vector, G0, G1, and G2 (grown on coverslips) were co-labeled for F-actin (by phalloidin) and dynamin (n = 3). Nuclei were stained with DAPI. Cells were examined under a fluorescence microscope. Representative fluoromicrographs are shown in Fig. 4J. DPDs expressing vector and G0 displayed well- spread and intact actin filaments. On the other hand, DPDs expressing G1 and G2 displayed predominantly cortical labeling for F-actin and a decreased number of actin filaments. Similarly, DPDs expressing vector and G0 displayed moderate expression of dynamin. However, DPDs expressing G1 and G2 displayed minimal expression of dynamin. Accumulation of F-actin at the cortices of phalloidin labeled cells in vector- and APOL1G0-, APOLG1-, and APOL1G2-expressing cells were graded in the form of cortical F-actin score index. Cumulative data is shown in a bar diagram (Fig. 4K). DPDs expressing APOL1G1 and APOL1G2 displayed a higher (P < 0.01) cortical actin score when compared to DPDs expressing Vector or APOL1G0.

### APOL1G0 binds with one of the constituents of the ACs, but APOL1 risk alleles lack this property

**Figure 5:**
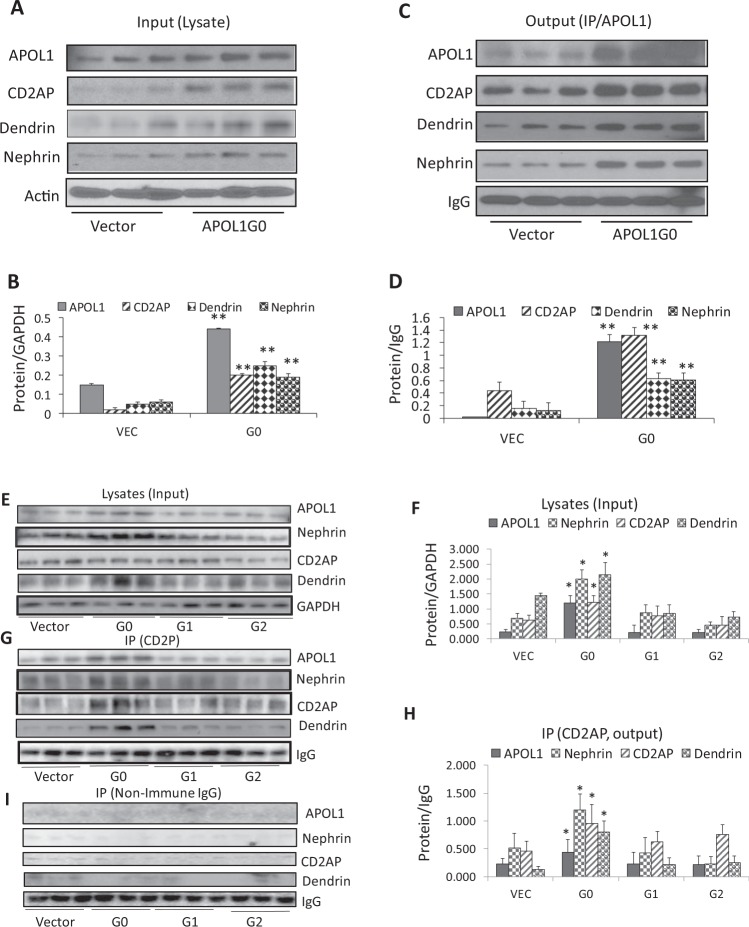
APOL1 binds with one of the constituents of adherens complex. (**A**) Cellular lysates of DPDVs (DPDs expressing vector) and DPDG0s (DPDs expressing APOL1 G0) were probed for APOL1, CD2AP, dendrin, nephrin, and reprobed for actin (n = 3). Gels from three different lysates are displayed. (**B**) Cumulative densitometric data from protein blots displayed in 5A. **P < 0.01 compared with respective vector variables. (**C**) The cellular lysates of DPDVs and DPDG0s in the 5A were immunoprecipitated (IP) with an anti-APOL1 antibody (n = 3). IP fractions were probed for APOL1, CD2AP, dendrin, nephrin, and IgG. Gels from three different fractions are displayed. (**D**) Cumulative densitometric data from protein blots displayed in 5C. **P < 0.01 compared to respective Vec. (**E**) The cellular lysates of DPDVs, DPDG0s, DPDG1s, and DPDG2s were probed for APOL1, nephrin, CD2AP, CTSL, dynamin, and GAPDH (n = 3). Gels from three different lysates are displayed. (**F**) Cumulative densitometric data from protein blots displayed in 5E. *P < 0.05 compared to respective Vec, G1, and G2. (**G**) The cellular lysates in the 5E were immunoprecipitated with an anti-CD2AP antibody. IP fractions of three different lysates were probed for APOL1, CD2AP, dendrin, nephrin, and IgG. Gels from there different IP fractions have been displayed. (**H**) Cumulative densitometric data from protein blots displayed in 5G. *P < 0.05 compared to respective Vec, G1, and G2. (**I**) The cellular lysates of DPDVs, DPDG0s, DPDG1s, and DPDG2s in the 5E were immunoprecipitated (IP) with a non-specific mouse IgG (n = 3). IP fractions were probed for APOL1, CD2AP, dendrin, nephrin, and IgG. Gels from three different fractions are displayed. For clarity lanes were cropped from different gels. Full-length blots are presented in Fig. S5.

To evaluate whether constituents of the ACs are present in DPDV (DPDs-stably expressing vector) and DPDG0 (DPDs-stably expressing ectopic APOL1G0), cellular lysates of DPDVs and DPDG0s were probed for APOL1, CD2AP, dendrin, nephrin and reprobed for GAPDH (n = 3). Gels from three different lysates are displayed in Fig. 5A. Densitometric data are shown in Fig. 5B. DPDG0 displayed enhanced expression of APOL1, CD2AP, dendrin, and nephrin when compared to DPDVs.

To determine the binding status, cellular lysates of DPDVs and DPDG0s were immunoprecipitated (IP) with an anti-APOL1 antibody (n = 3). IP fractions were probed for APOL1, CD2AP, dendrin, nephrin, and IgG. Gels from three different fractions are displayed in Fig. 5C. Densitometric data is shown in Fig. 5D. IP fractions of DPDG0 displayed enhanced expression of APOL1, CD2AP, dendrin, and nephrin. These findings suggest that APOL1 binds with CD2AP along with other constituents of the ACs.

To determine the status of APOL1 binding in DPDs expressing APOL1 risk alleles, cellular lysates of DPDVs, DPDG0s, DPDG1s, and DPDG2s were probed for APOL1, nephrin, CD2AP, dendrin, and GAPDH (n = 3). Gels from three different lysates are displayed in Fig. 5E. Densitometric data are shown in Fig. 5F. DPDG1s and DPDG2s displayed decreased expression of nephrin, CD2AP, and dendrin when compared to DPDG0s. These findings suggest destabilization of ACs in DPDs expressing APOL1 risk alleles.

To substantiate this, the above-mentioned cellular lysates were immunoprecipitated with an anti-CD2AP antibody. IP fractions from different lysates were probed for the constituents of the ACs and IgG. Gels from three different IP fractions have been shown in Fig. 5G. Cumulative densitometric data are shown in bar graphs (Fig. 5H). DPDG0s displayed a higher expression for APOL1, nephrin, CD2AP, and dendrin when compared to DPDG1s and DPDG2s. These findings confirm the occurrence of the destabilization of the complexes in DPDs expressing G1 and G2.

To exclude the non-specific binding of IgG to APOL1 or CD2AP, lysates of DPDV, DPDG0, DPDG1, and DPDG2 were immunoprecipitated with non-immune IgG. IP fractions were probed for APOL1, CD2AP, dendrin, and IgG. Gels from three different lysates are shown in Fig. 5I.

### APOL1 risk alleles induce disruption in the APOL1-miR193 axis

**Figure 6:**
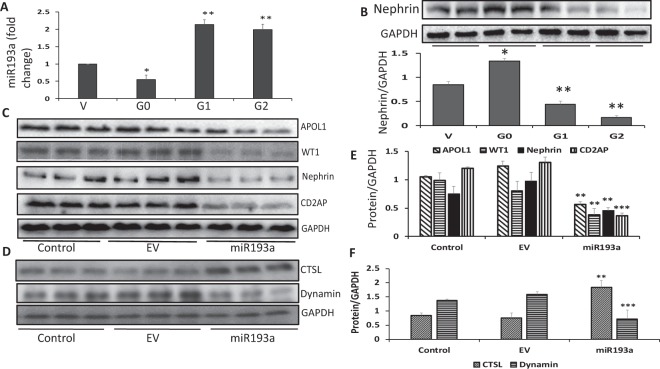
APOL1 risk alleles induce disruption in APOL1-miR193a axis. (**A**) RNAs were extracted from DPDs expressing vector, G0, G1, and G2 (n = 3). RNAs were assayed for miR193a. Cumulative data are shown in bar graphs. *P < 0.05 compared with V; **P < 0.01 compared with all other variables. (**B**) Protein blots of DPDs expressing vector, G0, G1, and G2 were probed for nephrin and reprobed for actin (n = 3). Gels from two different lysates are displayed in the upper panel. Cumulative densitometric data are shown as bar graphs in the lower panel. *P < 0.05 compared with V; **P < 0.01 compared with all other variables. (**C**) DPDs were transfected with either empty vector (25 nM, EV) or mir193a plasmid (25 nM). Protein blots of control and experimental DPDs (transfected with either EV or miR193a plasmid) were probed for APOL1, WT1, nephrin, CD2AP and reprobed for GAPDH. Gels from three different lysates are shown. (**D**) The lysates in 6C were probed for CTSL and dynamin and reprobed for GAPDH. Gels from three different lysates are shown. (**E**) Cumulative densitometric data (from 6C) are shown in bar graphs. **P < 0.01 compared with respective control and EV; ***P < 0.001 compared with respective control and EV. (**F**) Cumulative densitometric data (from D) are shown in bar graphs. **P < 0.01 compared with respective control and EV; ***P < 0.001 compared with respective control and EV. For clarity lanes were cropped from different gels. Full-length blots are presented in Fig. S6.

We asked whether DPD-expressing APOL1 risk alleles carry an intact APOL1-miR193a axis. To confirm the status of APOL1-miR193a axis in DPDs-stably expressing vector, G0, G1, and G2, RNAs were extracted and assayed for miR193a (n = 3). Cumulative data are shown in bar graphs in Fig. 6A. DPDs expressing G0 down-regulated the expression of miR193 by 50% when compared to DPDs expressing vector. However, DPDs expressing G1 and G2 showed upregulation (two-fold) of miR193a. These findings indicate that an intact (functional) APOL1-miR193a axis in DPDs requires the expression of non-risk APOL1.

Since miR193a inversely regulates the expression of WT1^22^, a transcription factor for the expression of nephrin, disruption of APOL1-miR193a axis would result in suboptimal transcription of nephrin in DPDs expressing APOL1G1 and APOL1G2. To validate our notion, protein blots of DPDs expressing vector, G0, G1, and G2 were probed for nephrin and reprobed for GAPDH (n = 3). Gels from two different lysates are displayed in Fig. 6B (upper panel). DPDs expressing APOL1 risk alleles showed a decreased expression of nephrin. Cumulative densitometric data are shown as bar graphs (Fig. 6B, lower panel).

We asked if APOL1 risk alleles are inducing destabilization of ACs as a consequence of the upregulation of miR193a expression. If so then a higher level of miR193a *de novo* would down-regulate WT1, nephrin, and CD2AP in DPDs. To validate the role of the miR193a-APOL1 axis, DPDs were transfected with either empty (EV) vector or miR193a plasmid. Protein blots of control and experimental DPDs (transfected with either EV or miR193a plasmid) were probed for APOL1, WT1, nephrin, CD2AP and reprobed for GAPDH. The same lysates were reprobed for CTSL, dynamin, and GAPDH. Gels from three different lysates are shown in Fig. 6C and D. Cumulative densitometric data are shown in bar graphs (Fig. 6E and F). An overexpression of miR193a in DPDs, downregulated APOL1, WT1, nephrin, CD2AP, dynamin, but upregulated the expression of CTSL. These findings suggest that APOL1 risk alleles down-regulate the expression of at least one of the constituents of ACs through disruption of the APOL1-miR193a axis.

### Vitamin D receptor agonist (VDA) down-regulates miR193a and modulates APOL1 risk alleles-induced downstream signaling

**Figure 7:**
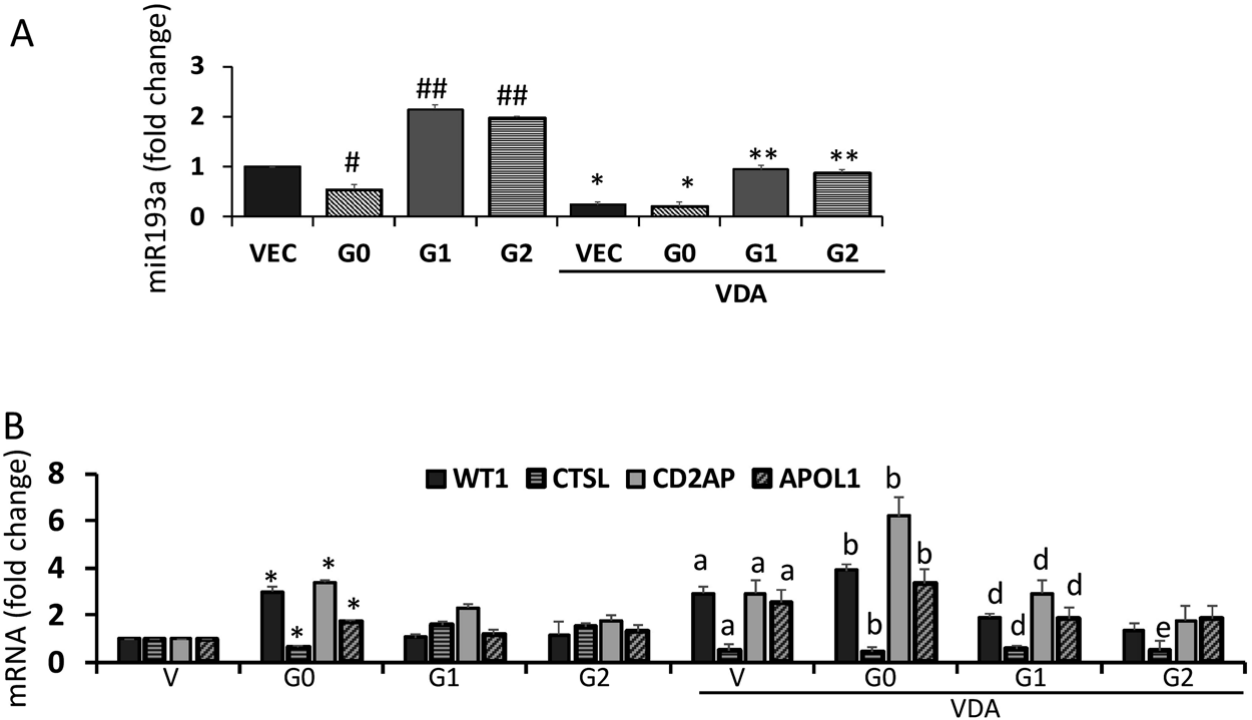
Vitamin D receptor agonist (VDA) down-regulates miR193a and modulates APOL1 risk-induced downstream signaling. (**A**) DPDVs (VEC), DPDG0s (G0), DPDG1s (G1), and DPDG2s (G2) were incubated in media containing either vehicle (DMSO) or VDA (EB1089, 10 nM) for 48 hours. RNAs were extracted and assayed for miR193a (n = 3). Cumulative data are shown in bar graphs. ^#^P < 0.05 compared VEC; ^##^P < 0.01 compared with VEC and G0. *P < 0.05 compared with untreated VEC and G0; **P < 0.01 compared with untreated G1 and G2. (**B**) RNAs were extracted from DPDVs (V), DPDG0s (G0), DPDG1 (G1), and DPDG2s (G2) and cDNAs were amplified with specific primers for *APOL1*, *WT1*, *CD2AP*, and *CTSL* (n = 3). Cumulative data are shown as bar graphs. *P < 0.05 compared to respective V; ^a^P < 0.05 compared to respective V; ^b^P < 0.05 compared to respective G0; ^d^ < 0.05 compared to respective G1; ^e^P < 0.05 compared to respective G2.

To determine whether VDA would be able to resolve APOL1 risk alleles-induced disruption of the APOL1-miR193a axis, DPDVs, DPDG0s, DPDG1s, and DPDG2s were incubated in media containing either vehicle (DMSO) or VDA (EB1089, 10 nM) for 48 hours. RNAs were extracted and assayed for miR193a (n = 3). Cumulative data are shown in bar graphs (Fig. 7A). In DPDG0, expression of miR193a was down-regulated while in DPDG1 and DPDG2, it was enhanced. VDA down-regulated miR193a expression in DPDV, DPDG0s, DPDG1s, and DPDG2s by several folds when compared to respective controls.

To determine the effect of VDA on the transcription of *APOL1*, *WT1*, *CD2AP*, and *CTSL*, RNAs were extracted from DPDVs, DPDG0s, DPDG1s, and DPDG2s. cDNAs were amplified with specific primers for *APOL1*, *WT1*, *CD2AP*, and *CTSL* (n = 3). Cumulative data are shown in bar graphs in Fig. 7B. VDA enhanced the transcription of *APOL1*, and *CD2AP*, but decreased the transcription of *CTSL* in DPDVs, DPDG0s, DPDG1s, and DPDG2s.

### VDA down-regulates Puromycin aminonucleoside (PAN)-induced DPD expression of miR193a

**Figure 8:**
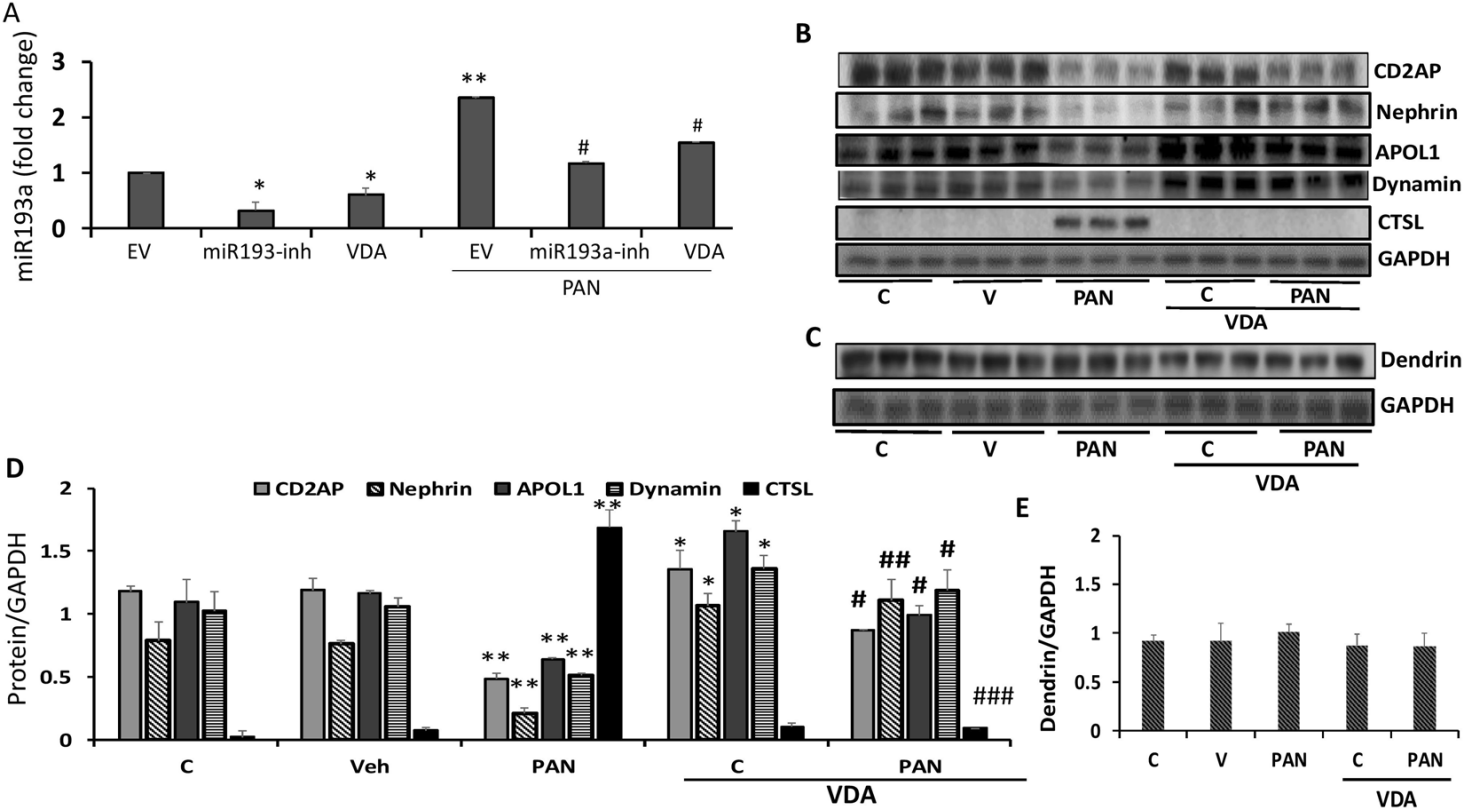
VDA down regulates PAN-induced DPD expression of miR193a. (**A**) DPDs were transfected with either empty vector (EV) or miR193a inhibitor (plasmid, 25 nM), or treated with VDA (EB1089, 10 nM) in the presence or absence of PAN (30 ng/ml) for 48 hours (n = 3). RNAs were extracted and assayed for miR193a. Cumulative data are shown in bar graphs. *P < 0.05 compared with respective EV. **P < 0.01 compared with untreated EV; ^#^P < 0.01 compared with PAN-treated EV. (**B**) DPDs were incubated in media containing either buffer (Control, C), vehicle (Veh, DMSO), or PAN (30 ng/ml) with or without VDA (EB1089, 10 nM) for 48 hours (n = 3). Protein blots were probed for CD2AP, nephrin, APOL1, dynamin, and CTSL. The same blots were reprobed for GAPDH. Gels from three different lysates are shown. (**C**) The lysates in 8B were probed for dendrin (n = 3). Gels from three different lysates are displayed. (**D**) Cumulative densitometric data from the blots of 8B are shown in bar graphs. *P < 0.05 compared to respective C and Veh; **P < 0.05 compared to respective C and Veh; ^#^P < 0.05 vs. PAN alone; ^##^P < 0.01 vs. PAN alone; ^###^P < 0.001 vs. PAN alone. (**E**) Cumulative densitometric data from the blots of 8C are shown in bar graphs. For clarity lanes were cropped from different gels. Full-length blots are presented in Fig. S8.

PAN is known for its podocyte cytotoxicity^5,20^. We asked whether PAN enhances DPDs’ expression of miR193a; if yes, whether VDA or miR193a inhibitor would be able to down-regulate PAN-induced miR193a expression. To determine the effect of VDA and a miR193a inhibitor on PAN-induced miR193a expression, DPDs were transfected with either empty vector (EV) or miR193a inhibitor (plasmid, 25 nM), or treated with VDA (EB1089, 10 nM) in the presence or absence of PAN (30 ng/ml) for 48 hours (n = 3). RNAs were extracted and assayed for miR193a. Cumulative data are shown in bar graphs (Fig. 8A). PAN enhanced DPDs’ expression of miR193a by 2.5 fold. Both miR193a inhibitor and VDA down-regulated (P < 0.05) DPDs’ expression of miR193a in PAN milieu.

To determine the effect of VDA on PAN-modulated ACs and associated downstream effects, DPDs were incubated in media containing either vehicle (Veh, DMSO) or PAN (30 ng/ml) with or without VDA (EB1089, 10 nM) for 48 hours (n = 3). Protein blots were probed for CD2AP, nephrin, APOL1, dynamin, and CTSL. The same blots were reprobed for GAPDH. The same lysates were also probed for dendrin and GAPDH. Gels from three different cellular lysates are shown in Fig. 8B and C. Cumulative densitometric data are shown in bar graphs (Fig. 8D and E). PAN down-regulated the expression of nephrin, APOL1, dynamin, CD2AP, and dynamin but enhanced the expression of CTSL. These findings suggest that PAN destabilizes the complex. On the other hand, VDA protects against these effects of PAN.

### Nuclear import of dendrin in podocytes in APOL1G1 transgenic mice

**Figure 9:**
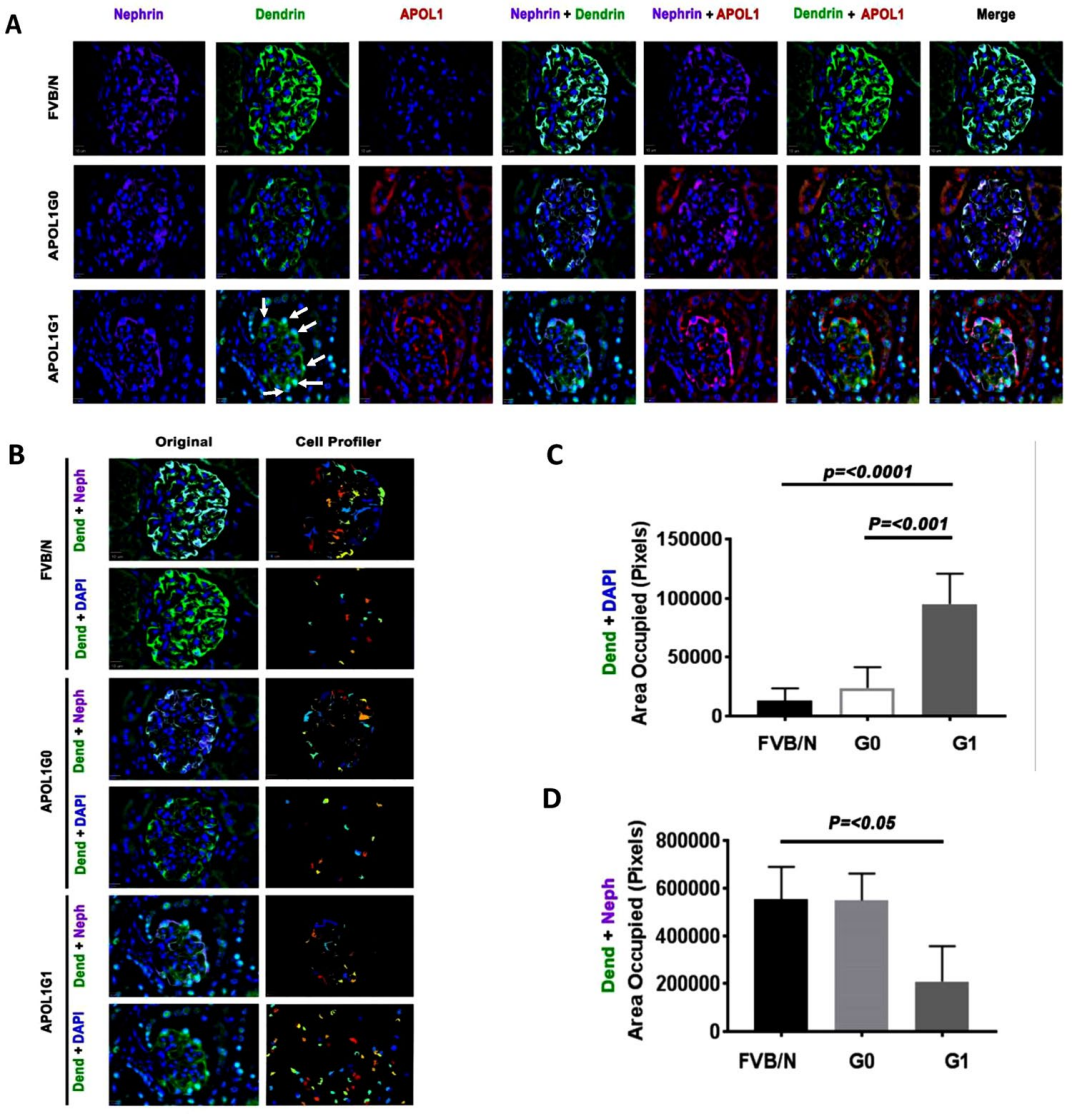
Nuclear import of dendrin in podocytes in APOL1G1 transgenic mice. (**A**) Renal cortical sections of FVB/N, APOL1G0 (G0), and APOL1G1 (G1) transgenic mice (n = 4) were labeled for nephrin, APOL1, and dendrin. Representative microphotographs are displayed. Podocytes of an FVB/N mouse showed cytosolic labeling for nephrin (purple fluorescence) and dendrin (green fluorescence). Tubular cells also showed labeling for dendrin. Podocytes in an APOL1G0 transgenic mouse showed cytosolic labeling for APOL1 (red fluorescence), nephrin (purple fluorescence), and dendrin (green fluorescence). Tubular cells also showed labeling for APOL1 (red fluorescence). On the other hand, an APOLG1 transgenic mouse showed predominantly nuclear import of dendrin (green fluorescence, indicated by white arrows). Original Mag.X400. (**B**) A pipeline of modules of Broad Institute’s CellProfiler suite was used to analyze dendrin expression in podocytes. Representative original images of FVB/N, APOL1G0 and APOL1G1 glomeruli showing expression of nephrin (purple) dendrin (green) and DAPI (blue) were captured using Slidebook 6.0 software. Images were then processed using Cell Profiler pipeline to analyze expression of dendrin in cytosol and nucleus. Dendrin expressing pixels co-labeled with either nephrin or DAPI were measured and the total area occupied was calculated. Processed images are showing randomly denoted colors of the area occupied by pixels expressing dendrin and nephrin or dendrin and DAPI. Mag.X400. (**C**) Randomly selected glomeruli from FVB/N, APOL1G0 and APOL1G1 mice (Fig. 9B) were analyzed and data of the area occupied by pixels expressing dendrin and DAPI was collected. A bar graph is showing the number of pixels co-expressing dendrin and DAPI. (**D**) Randomly selected glomeruli from FVB/N, APOL1G0 and APOL1G1 mice (Fig. 9B) were analyzed and data of the area occupied by pixels expressing dendrin and nephrin was collected. A bar is showing the number of pixels co-expressing dendrin and nephrin.

Four-week-old FVB/N, APOL1^*G0*/*G0*^, and APOL1^*G1*/*G1*^ transgenic mice (n = 4) were fed doxycycline in their feed for four weeks. Subsequently, kidneys were harvested, and renal cortical sections were labeled for nephrin, APOL1, and dendrin. Representative fluoromicrographs are shown in Fig. 9A. Podocytes of FVB/N mice showed cytosolic labeling for nephrin (purple fluorescence) and dendrin (green fluorescence). Tubular cells also showed labeling for dendrin. Podocytes in APOL1^*G0*/*G0*^ transgenic mice showed cytosolic labeling for APOL1 (red fluorescence), nephrin (purple fluorescence), and dendrin (green fluorescence). Tubular cells also showed labeling for APOL1 and dendrin. On the other hand, APOL1^*G1*/*G1*^ transgenic mice showed predominantly nuclear labeling for dendrin (green) in their podocytes. Dendrin partially co-labeled with DAPI (Fig. 9C) or nephrin (9D) were quantified using the Cell Profiler Program (Fig. 9B). These findings suggest that APOL1G1 induces nuclear import of dendrin in podocytes *in vivo*.

### Albuminuria and glomerular sclerotic lesions in APOL1^*G1*/*G1*^ and APOL1^*G1*/*G2*^ mice

**Figure 10:**
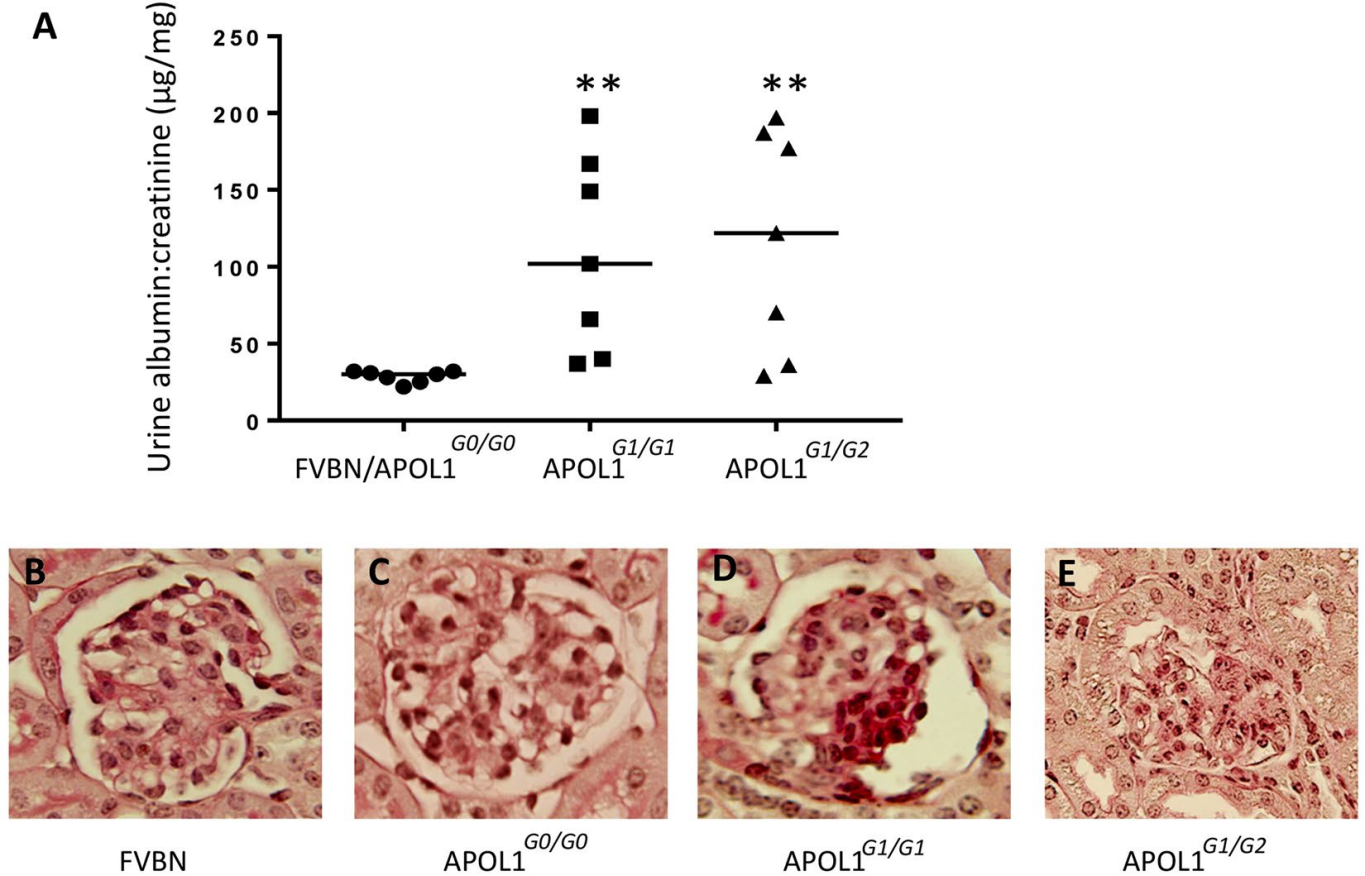
Albumin to creatinine ratios and glomerular lesions in mice. (**A**) Four-week-old control (FVBN, n = 4) and transgenic mice (APOL1^*G0*/*G0*^, n = 3; APOL1^*G1*/*G1*^, n = 7); APOL1^*G1*/*G2*^, n = 7) were fed doxycycline in their feed. After six weeks urine was collected and albumin to creatinine ratio was assayed. Value of each mouse (urine sample) is shown in the dot plot. **P < 0.01 compared with FVBN/APOL1^*G0*/*G0*^. (**B**) A representative glomerulus from an FVBN mouse. Mag.X400. (**C**) A representative glomerulus from an APOL1^*G1*/*G1*^ mouse showing segmental solidification and expansion of the mesangial area with increased matrix and cellularity starting at the vascular pole and obliterated capillary space, findings consistent with FSGS. A focal adhesion of the capillary tuft is also present at about 2 o ’clock position. Mag.X400. (**D**) A representative glomerulus from an APOL1^*G1*/*G2*^ mouse showing a larger area of segmental solidification and expansion of the mesangium at 3 to 5 o’ clock position compared to APOL1^*G1*/*G1*^ associated with obliterated capillary spaces, findings consistent with FSGS. A focal adhesion of the capillary tuft is also present at about 5 o ’clock position. Mag.X400.

APOL1^*G0*/*G0*^ mice did not show any increase in urinary albumin to creatinine ratio when compared to FVBN (28.57 ± 1.4 vs. APOL1^*G0*/*G0*^, 29.1 ± 1.2). APOL1^*G1*/*G1*^ (108.4 ± 24.3) and APOL1^*G1*/*G2*^ (116.9 ± 27.4) transgenic mice showed an increase (P < 0.01) in urinary albumin to creatinine ratio. However, only four out of seven (57%) of APOL1^*G1*/*G1*^ and APOL1^*G1*/*G2*^ mice developed albuminuria (4-fold increase in albumin: creatinine ratio when compared to FVBN and APOL1^*G0*/*G0*^). Urinary albumin to creatinine ratio in each mouse is shown in Fig. 10A.

FVBN, APOL1^*G0*/*G0*^ and APOL1^*G1*/*G1*^ and APOL1^*G1*/*G2*^ mice with normal renal histology displayed normal urinary albumin to creatinine ratio. APOL1^*G1*/*G1*^ and APOL1 ^*G1*/*G2*^ transgenic mice with abnormal urine to creatinine ratio showed segmental solidification and sclerotic lesions in 15 to 18% of glomeruli. Representative microphotographs are shown in Fig. 10B.

**Figure 11:**
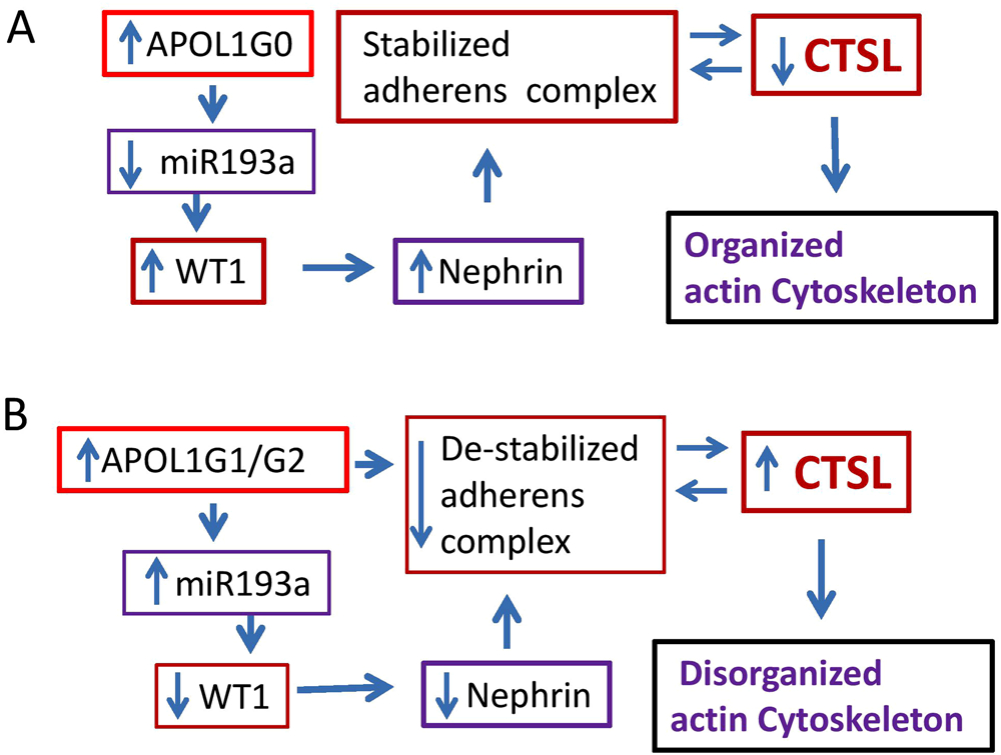
Proposed hypothetical schemes. (**A**) APOL1G0 preserves actin cytoskeleton through down-regulation of miR193a and stimulation of nephrin expression, one of the constituents of the adherens complex. (**B**) APOL1G1 and APOL1G2 disrupt APOL1-miR193a axis and induce upregulation of miR193a resulting down-regulation of WT1 and associated attenuated expression of nephrin, one of the constituents of the complex.

A proposed formulation to integrate these findings is schematically demonstrated in Fig. 11. Ectopic overexpression of APOL1G0 in PDs down regulates miR193a expression resulting in an upregulation of WT1 and nephrin, which stabilizes the AC (Fig. 11A). The absence of nuclear dendrin decreases CTSL levels and thus prevents degradation of CD2AP, dynamin, and synaptopodin, the molecules which preserve the actin cytoskeleton in podocytes (Fig. 11A). On the other hand, DPDs expressing APOL1G1 and APOL1G2 are defective in down-regulation of miR193a, consequently resulting in attenuated expression of WT1 and nephrin that destabilizes the ACs and enhances transcription of CTSL; the latter induces disorganization of the actin cytoskeleton in podocytes (Fig. 11B).

## Discussion

In the present study, overt expression of APOL1G0 in podocytes, down-regulated the expression of miR193a indicating an intact APOL1 miR193a axis. The expression of APOL1G1 and G2 in DPDs was associated with upregulation of miR193a suggesting a disruption in APOL1-miR193a axis. DPDG0 showed a robust expression, but DPDG1s and DPDG2s displayed attenuated expression of the AC constituents. Enhanced expression of CTSL and down-regulation of dynamin expression also suggested the destabilization of the ACs in DPDG1s and DPDG2s. Downstream effects of the destabilized ACs manifested in the form of nuclear import of dendrin and disorganization of the actin filaments in DPDG1s and DPDG2s. Since DPDs overexpressing miR193a also showed down-regulation of the AC constituents and upregulation of CTSL, it appears that disruption of APOL1-miR193a axis contributed to the destabilization of the ACs and disorganization of the actin filaments in DPDG1s and DPDG2s. *In vivo* studies, APOL1^*G1*/*G1*^ and APOL1^*G1*/*G2*^ transgenic mice exhibited nuclear import of dendrin, a consequence of destabilized ACs in podocytes. APOL1^*G1*/*G1*^ and APOL1^*G1*/*G2*^ transgenic mice also showed a four-fold increase in proteinuria when compared to control and APOL1^*G0*/*G0*^ transgenic mice. Renal histology revealed the development of segmental sclerotic lesions in 15–18% of glomeruli in APOL1 ^G1/G1^ and APOL1^*G1*/*G2*^ transgenic mice. PAN enhanced, but VDA inhibited the expression of miR193a in podocytes in PAN-stimulated state. A miR193a inhibitor and VDA provided protection against APOL1 risk alleles- and PAN-induced down-regulation of the transcription of the AC constituents in podocytes. Since enhanced miR193a expression down-regulated nephrin expression, miR193a could have destabilized the ACs and disorganized the actin cytoskeleton in adverse milieus as well as in mutant podocytes.

Interestingly, imaging studies in DPDG0s showed co-labeling of APOL1 and CD2AP at the adherent junctions. Docking studies also showed binding of APOL1 to CD2AP; these findings suggest an interaction between APOL1 and CD2AP. However, the role of this interaction in the stabilization of the ACs is not clearly understood to date.

The net outcome of the silencing of APOL1 as well as of an overt expression of APOL1 risk alleles were similar i.e., increased transcription of the *CTSL*. Since APOL1 inversely regulates miR193a^19^, the lack of APOL1 would increase PD expression of miR193a. Also, overt expression of APOL1 risk alleles elevated miR193a levels in DPDs. In both the instances, miR193a would down-regulate the expression of nephrin resulting in destabilization of ACs and associated increased transcription of CTSL. Thus, it appears that expression of APOL1 risk alleles is equivalent to the loss of the function of APOL1G0 in podocytes.

Interestingly, miR193a down-regulates APOL1 expression in podocytes^19^. Does an increase in miR193a expression represent an attempt to down-regulate APOL1 risk alleles by podocytes? In that scenario, miR193a-mediated toxicity would be considered as a gain of function by APOL1 risk alleles.

CD2AP and CTSL have been reported to have a feedback relationship in podocytes^27^. Podocyte injury down-regulates the expression of CD2AP culminating in the destabilization of the ACs and enhanced transcription of CTSL^27^. The latter augments the degradation of CD2AP, which in turn would destabilize the ACs and result in the nuclear import of dendrin and associated transcription of CTSL. In the present study, enhanced expression of APOL1 contributed to an increased expression of CD2AP and a decreased expression of CTSL; conversely, silencing of APOL1 in DPDs down-regulated the expression of CD2AP and enhanced the expression of CTSL. Therefore, our observations regarding CD2AP and CTSL relationship are consistent with the findings of the other investigators^27^.

In earlier studies, the loss of any of the components of the ACs led to the activation of the apoptotic pathway^24^. These mechanistic studies served as the basis for the loss of glomerular filtration barrier in CD2AP and nephrin knockout mice. In our present study, silencing of nephrin also destabilized the ACs in podocytes. As expected, an elevation of miR193a in podocytes as a consequence of silencing of APOL1 or their mutation not only down-regulated nephrin expression but also destabilized the AC. Additionally, elevated miR193a levels (as a consequence of APOL1 silencing) could have down-regulated CD2AP expression through enhanced CTSL expression and exacerbation of autophagy^33^. Whether APOL1 silencing-induced loss of APOL1-CD2AP interaction has affected the stability of the ACs independent of its effect on miR193a expression is not clear.

In the present study, PAN destabilized the ACs in DPDG0s. Since PAN enhanced miR193a expression, it appears that PAN could have destabilized the ACs through down-regulation of nephrin. Notably, VDA is known to down-regulate miR193a expression in PDs^19,23^. As expected, VDA provided protection against PAN-induced destabilization of the ACs and associated downstream effects through down-regulation of miR193a. These findings indicate that down-regulation of miR193a plays an important role in the stabilization of the ACs. Therefore, this strategy could be used as a therapeutic tool to protect against podocyte injury induced by adverse milieus associated with elevated levels of miR193a.

We have earlier reported that overexpression of APOL1G1 and G2 induced lysosomal leakage of CTSL and interruption of the actin cytoskeleton in podocytes^5^. In those studies, we proposed the gain of function by APOL1 risk alleles. The present study demonstrates that an optimal expression level of APOL1G0 maintains a low level of CTSL in the cytosol in PDs. It is conceivable in addition to pathways for a gain of function injury previously reported; there may also be a contribution to elevated cytosolic levels of CTSL and associated downstream effects in PDs expressing APOL1G1 and G2 due to a loss of function related to the maintenance of the ACs. The interrelation and the relative contribution of these mechanisms require further investigation and may also impinge on drug discovery strategies that target APOL1 risk alleles. Since organisms, which do not possess or express APOL1, seem to preserve the integrity of podocytes, this indicates that there are perhaps other mechanisms for the preservation of the complex. More specifically, podocyte ACs have been demonstrated to be functionally active in mice even though they lack APOL1^21,27^. That suggests it is not the APOL1, but rather disruption in the APOL1-miR193a axis which is contributing to the destabilization of the ACs. The role of miR193a in the induction of injury in mouse podocytes has been demonstrated both *in vitro* and *in vivo* studies^22^. *In vivo* studies, miR193a transgenic mice demonstrated loss of WT1, nephrin, and CD2AP in podocytes^22^. These mice eventually developed focal segmental glomerulosclerosis. Thus, it appears that levels of miR193a have to be relatively lower to preserve podocyte molecular phenotype in mice.

The question arises if APOL1 is not required for the regulation of miR193a axis in the majority of the species, why have humans evolved APOL1 as a part of this axis? One explanation could be the shorter lifespan of mice and other species meaning thereby that they do not require highly specialized mechanisms for long-lived maintenance for terminally differentiated PDs. One may conjecture that the lack of APOL1 in mice could make them relatively more susceptible to podocyte injury. On the other hand, human beings are longer living mammals, requiring sustained longevity for terminal differentiated podocytes. To accomplish that goal, human PDs require extra safeguards for their protection. Nonetheless, the notion that evolution of APOL1 in human beings as a constituent of miR193a axis to sustain longevity to PDs remains conjecture at present.

We conclude that an intact APOL1-miR193a axis plays an important role in the maintenance of the integrity of actin cytoskeleton, whereas, its disruption in podocytes expressing APOL1 risk alleles make them susceptible to disorganization of actin filaments.

## Material and Methods

### Human podocytes

Human podocytes (PDs) were conditionally immortalized by introducing temperature-sensitive SV40-T antigen by transfection^34^. DNA sequencing of these podocytes revealed the endogenous APOL1G0 genotype.

### Generation of stable cell lines expressing *APOL1G0*/*G1*/*G2* and Vector

Stable cell lines ectopically expressing *APOL1G0*, *APOL1G1*, *and APOL1G2 were* generated by retroviral infection in a different immortalized undifferentiated human podocyte cell line as described previously^15,19,35^.

Undifferentiated PDs expressing vector (PDV) or ectopic APOL1G0/G1/G2 were seeded on collagen-coated plates and differentiated through incubation in normal RPMI (containing 11 mM glucose) for ten days at 37 °C (DPDVs, DPDG0s, DPDG1s, and DPDG2s). Since DPDG0s, DPDG1, and DPDG2 displayed unusually high expression of APOL1, the protein loading was decreased to reduce the expression; however, a decrease in protein loading minimized the APOL1 expression in DPDVs as described in our previous publication^15^.

### Generation and Identification of APOL1^*G0*/*G0*^, APOL1^*G1*/*G1*^, and APOL1^*G1*/*G2*^ Transgenic Mice

The TetOn3G-APOL1G0 construct was released from the plasmid vector backbone by digestion with XhoI and purified by gel electrophoresis, and DNA was extracted using a QIAEX II Gel Extraction Kit (Qiagen). The purified DNA construct was introduced into the pronuclear of fertilized oocytes (from the FVB/N mouse) by microinjection using standard techniques. Details on characteristics of these mice have been described in our recent publication^36^. All experimental protocols were approved by the local committee for laboratory animal welfare of the Feinstein Institute for Medical Research, Manhasset, New York. All experiments were performed by the institutional guideline and regulations.

### Urine Albumin: creatinine ratios and renal histology in control and APOL1 transgenic mice

Four-week-old control (FVBN, n = 4), APOL1^*G0*/*G0*^ (n = 3), APOL1^*G1*/*G1*^ (n = 7), and APOL1^*G1*/*G2*^ (n = 7) were given doxycycline (200 mg/Kg) in their feed for six weeks. Subsequently, urine was collected, and kidneys were harvested. Albumin: creatinine ratio was assayed by an ELISA kit (Exocell Inc. Philadelphia). Renal cortical sections were stained with PAS. Ten glomeruli in each mouse were examined for the presence of glomerular sclerotic lesions.

### Transfection of miR193a inhibitor

miR193a inhibitor (25 nM; Cat #4464084; Thermo Fisher) and empty vector (25 nM; pCMV-MIR; Origene) were transfected in the cells using Lipofectamine 3000 Transfection Reagent (Thermo Fisher) according to the manufacturer’s protocol.

### Silencing of APOL1 and nephrin

DPDs were transfected with scrambled siRNA (control), APOL1 siRNA (20 nM; Santa Cruz), or nephrin siRNA (25 nM; Santa Cruz) with Lipofectamine RNAiMAX transfection reagent according to the manufacturer’s protocol (Thermo Fisher).

### RNA isolation and qPCR studies

Real-Time PCR was performed using one-step iTaq^TM^ Universal SYBR Green kit (BIO-RAD, USA) according to the manufacturer’s instructions using specific primers obtained from Thermo Fisher. *GAPDH* fw 5′ CCC ATC ACC ATC TTC CAG GAG 3′; rev 5′ GTT GTC ATG GAT GAC CTT GGC 3′, *WT1* fw 5′ CGAGAGCGATAACCACACAACG 3′; rev 5′ GTCTCAGATGCCGACCGTACAA 3′, CTSL fw 5′ GTTGCTATTGATGCAGGTCATGA 3′; rev 5′ ACTGCTACAGTCTGGCTCAAAATAAA ′3, APOL1 fw 5′ ATCTCAGCTGAAAGCGGTGAAC 3′; rev 5′ TGACTTTGCCCCCTCATGTAAG 3′, CD2AP fw 5′ CTGTCAGCTGCAGAGAAGAAA 3′; rev 5′ TTGGGTTGGAGAATGTCCAC 3′. Quantitative PCR was performed using an ABI Prism 7900HT sequence detection system and relative quantification of gene expression was calculated using the ΔΔCT method. Data were expressed as relative mRNA expression in reference to the control, normalized to the quantity of RNA input by performing measurements on an endogenous reference gene (GAPDH).

### MicroRNA assay

For miRNA quantification, the total RNA was isolated from control and experimental DPDs and miR193a assayed as described in our recent publications^19,36^.

### Immunofluorescence detection of F-actin and dynamin

Control and experimental podocytes were labeled for F-actin and dynamin as described in our previous publication^37^. In brief, cells were co-labeled with phalloidin (Alexa Fluor 568 #A12380; Thermo Fisher) and anti-dynamin antibody (mouse monoclonal; #sc-17807, Santa Cruz Biotechnology). DAPI was used for nuclear localization. Control and experimental cells were examined under immunofluorescence microscope.

The cortical F-actin score (CFS) index was determined based on three independent experiments (100 cells). The F-actin cytoskeletal reorganization for each cell was scored on a scale ranging from 0 to 3 based on the degree of cortical F-actin ring formation (score = 0, no cortical F-actin, normal stress fibers; score = 1, cortical F-actin deposits below one-half of the cell border; score = 2, cortical F-actin deposits exceeding one-half of the cell border; score = 3, complete cortical ring formatting and/or total absence of central stress fibers)^37^.

### Immunofluorescence of kidney tissue

Renal cortical sections from FVB/N (control), APOL1^*G0*/*G0*^ and APOL1^*G1*/*G1*^ transgenic mice were labeled with specific antibodies as described previously^36,37^. Primary antibodies (nephrin,rabbit polyclonal, 1:100 dilution, #ab58968; Abcam; APOL1, mouse monoclonal, 1;100 dilution, #66124-1-lg, Proteintech; dendrin, rabbit polyclonal, 1:100 dilution, #ab204787, Abcam) were used followed by the fluorescent conjugated secondary antibody. Slides were examined by confocal microscopy.

Images of dendrin, nephrin, and DAPI staining were captured on the same Z-axis using Slidebook 6.0 software. Image analysis and quantification were performed using Broad Institute’s (Cambridge, MA) CellProfiler suite^38^. Briefly, images were converted from the original color to gray and primary objects were identified as 20–60 pixel objects in DAPI channel and 5–200 pixel objects for dendrin and nephrin channels. Identified objects of dendrin were masked with the objects identified for nephrin to determine dendrin objects labeled with nephrin. Identified objects of dendrin were also masked with the objects identified for DAPI to determine dendrin objects labeled with DAPI. Binary outlines were created and the area occupied by dendrin labeled with nephrin or the area occupied by dendrin labeled with DAPI was measured^39^. All measurements were exported directly to CSV files and were subsequently analyzed using Graph Pad Prizm to generate bar graphs.

### Western blot studies

Western blot studies were carried out as described previously^5,6,19,37^. Primary antibodies against APOL1 (mouse monoclonal, 1:1000 dilution, Proteintech), WT1 (rabbit polyclonal, 1:1000 dilution, Abcam, MA), nephrin (rabbit polyclonal #ab58968; Abcam), CD2AP (rabbit polyclonal; #sc-25272; Santa Cruz), CTSL (rabbit polyclonal; #sc10778; Santa Cruz), dendrin (rabbit polyclonal, 1:1000 dilution, abcam), and dynamin (mouse monoclonal; #sc-17807, Santa Cruz) were used followed by treatment with horseradish peroxidase-labeled appropriate secondary antibodies. Equal protein loading and the protein transfer were confirmed by immunoblotting for determination of actin/GAPDH protein using a  monoclonal  β-Actin/GAPDH antibody (Santa Cruz, CA) on the same (stripped) western blots.

### Immunoprecipitation (IP)

Lysates from control and experimental PDs were immunoprecipitated with either an anti-APOL1 (mouse monoclonal, Proteintech) - or an anti-CD2AP antibody (rabbit polyclonal; sc-25272, Santa Cruz). IP fractions were probed for different constituents of ACs as described previously^19^.

### Protein-protein Interaction prediction

By sequence similarity and domain interactions, the prediction of a protein-protein interaction of APOL1 and CD2AP was made. Sequence similarity between proteins depends on the alignments from the basic local alignment search tool (BLAST)^40^. Further, STRING server version 10.0 was used as the STRING database intends to present a critical assessment and integration of protein-protein interactions, including direct (physical) as well as indirect (functional) alliances^41^.

### Homology Modeling of APOL1 and CD2AP

We retrieved the sequence of human Apolipoprotein 1 (APOL1) from the UniProt database (UniProt Id O14791). The sequence similarity with some of the templates was very low. Therefore, we selected two templates, i.e. c6ezvX (non-hemolytic enterotoxin lytic component l1) and c3gaab (protein from thermoplasma acidophilum) to model the APOL1 based on heuristics to maximize percentage identity, sequence coverage, and confidence using Phyre2 server^42^. Similarly, CD2AP (UniProt Id Q9Y5K6) was modeled using templates such as c2moxA (SH3 domain of sorbin and SH3 domain 2 containing protein 1) and c1griA (growth factor bound protein grb2).

### 3D modeling of miR193a

We retrieved the sequence of human miR193a-5p (miRBase Id = hsa-mir-193a-5p) from the miRBase database^43^. The 3D model of miR193a was generated using RNAComposer server, which is a method based on machine translation principle and operates on the RNA-FRABASE database^44^.

### Docking studies

Docking of APOL1G0 and CD2AP was performed using GRAMM-X web interface^45^ that uses the FFT (Fast Fourier Transformation) methodology by utilizing the smoothed potentials, refinement stage, and knowledge-based scoring. The docking of APOL1G0 and miR193a was performed using NPDock that combines GRAMM program to perform a rigid body global search, ranking and scoring of best decoys using statistical potentials, and clustering of best decoys; finally, to optimize the protein-nucleic acid interactions in the representative clusters, a Monte Carlo Simulated Annealing procedure (with protein and nucleic acid molecules treated as rigid bodies) was utilized^46^.

### Protein-protein and miRNA-protein interaction studies

The analyses of Protein-protein and miRNA-protein interactions were performed using SPPIDER^47^ and PDBePISA^32^ and KFC2^48^ servers. Visualization of PPI and miRNA-protein complexes was performed using PyMol^49^.

### Statistical analyses

Statistical comparisons were performed with the Prism program using the Mann–Whitney *U* test for nonparametric data and the unpaired *t* test for parametric data. A *P* value < 0.05 was considered statistically significant.

## Supplementary information

Supplementary data set
