# Supplementary material for: Disruption of APOL1-miR193a Axis Induces Disorganization of Podocyte Actin Cytoskeleton
Source: Sci Rep. 2019 Mar 5;9:3582. doi: 10.1038/s41598-019-39376-y (PMC6401370; doi:10.1038/s41598-019-39376-y)

Disruption of APOL1-miR193a Axis Induces Disorganization of Podocyte Actin Cytoskeleton

Vinod Kumar[1], Nitpriya Paliwal[1], Kamesh Ayasolla[1], Himanshu Vashistha[2], Alok Jha[1], Xiqian Lan[1], Nirupama Chandel[1], Sheetal Chowdhary[1], Moin A. Saleem[3], Ashwani Malhotra[1], Praveen N. Chander[4], Karl Skorecki[5], and Pravin C Singhal[1]

Figure 1.A

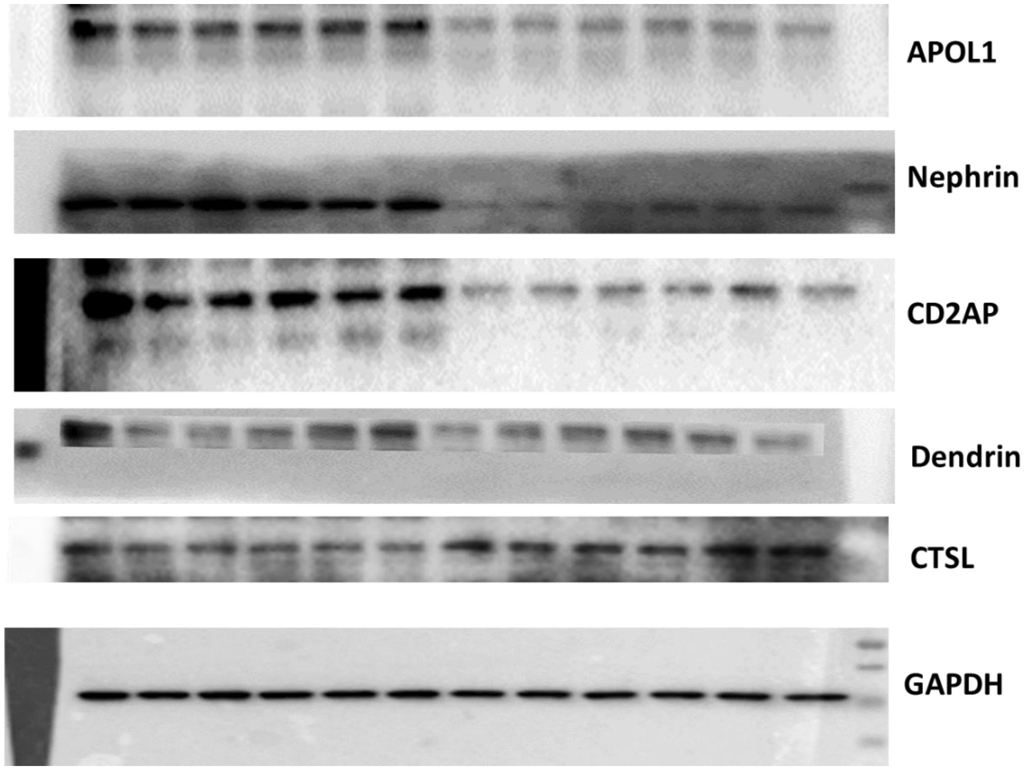

Figure 2.A

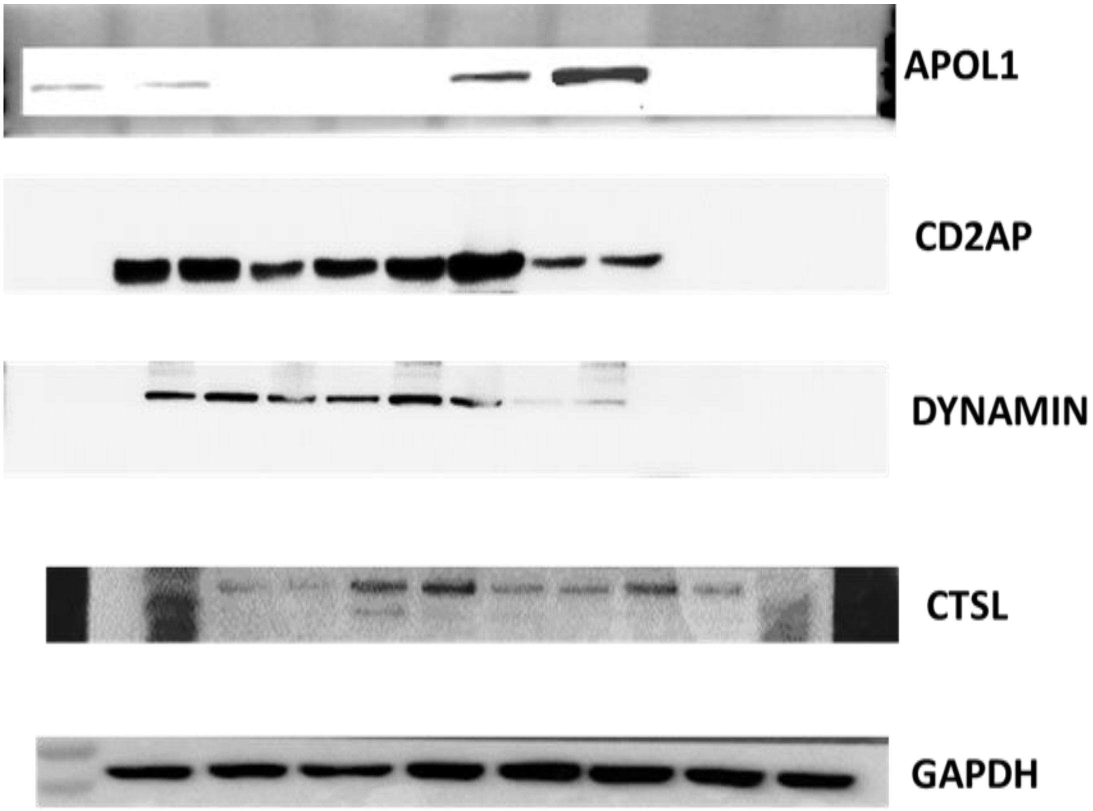

2.B

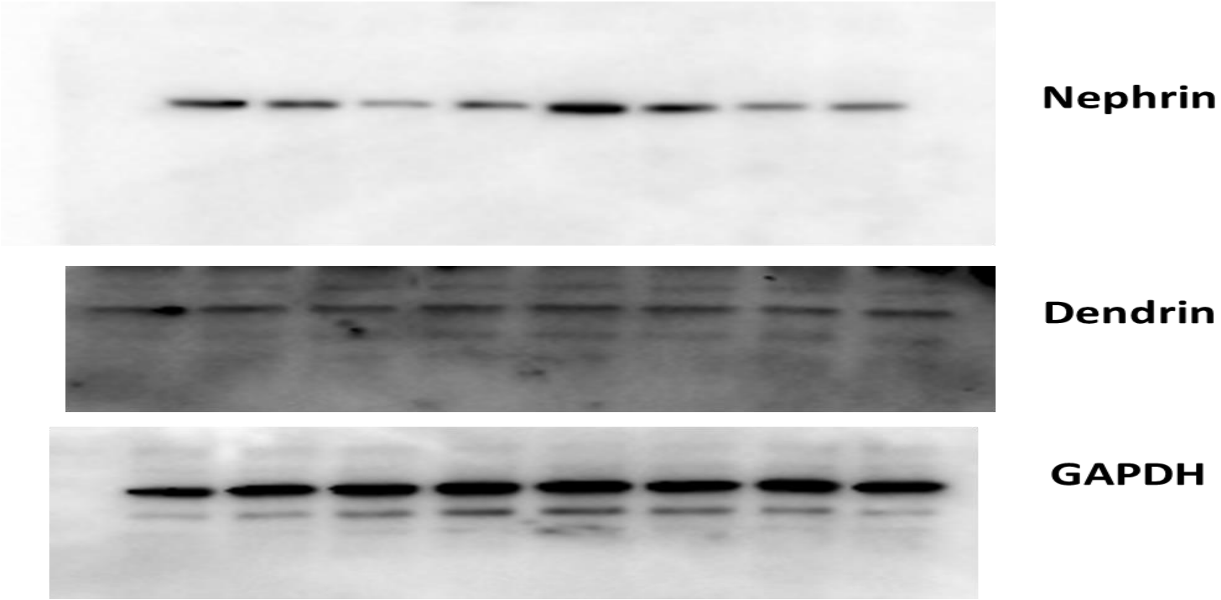

S. Fig. 3B

The PPI analyses from PDBePISA server (32) suggested that the APOL1-G0 and CD2AP complex has assemblies with multimeric state of 2 with formula AB, composition AB and the dissociation pattern is A+B. The surface area of the complex that indicates the total solvent-accessible surface area of the assembly in $Å^2$ is 56602.8 and the buried area that indicates the total solvent accessible area of the assembly, buried upon formation of all assembly's interfaces in $Å^2$ is 5346.7. ΔG^{int} that indicates the solvation free energy gain upon formation of the assembly in kcal/mol. is -36.3 kcal/mol. The value of ΔG^{diss}, which indicates the free energy of assembly dissociation in kcal/mol is 26.3 kcal/mol. The free energy of dissociation corresponds to the free energy difference between dissociated and associated states. Positive values of ΔG^{diss} indicate that an external driving force should be applied in order to dissociate the assembly, therefore, the assemblies with $\Delta G^{diss} > 0$ are thermodynamically stable. The rigid body entropy change at dissociation $T\Delta S^{diss}$ in kcal/mol is 15.3kcal/mol. The symmetry number that indicates the number of different but equivalent orientations of the assembly, which can be obtained by rotation, its value is 1 in the APOL1G0-CD2AP complex. The engaged interfaces have 2673.4 $Å^2$ buried area that indicates the interface area calculated as difference in total accessible surface areas of isolated and interfacing structures divided by two. The solvation free energy gain upon formation of interface $\Delta^i G$ is -36.3 kcal/mol. The value is calculated as difference in total salvation energies of isolated and interfacing structures. Negative $\Delta^i G$ corresponds to hydrophobic interfaces, or positive protein affinity. This value does not include the effect of satisfied hydrogen bonds and salt bridges across the interface. Also, there are 12 hydrogen bonds in the PPI interface of APOL1-G0 and CD2AP complex. We have also identified hotspot residues in the PPI interface of ApoL1-G0 and CD2AP that included Lys7, Ser10, Val11, Ile14, Met16,Glu92, Ala93, The172, Ala175, Asn176, Ser179, Gly180, Ser181, Leu182, Ser183, Ile184, Ser185, Gly187, Ile188, The190, Leu191, Val192, Gly193, Met194, Gly195, The200, Glu201, Leu208, Gly215, Ile216, Ala218, Leu220,The221, Ser225, Met228, Asp229, Arg303, Val306, The307, Ile310, Ser311, Ser314, Gly315, Gln317, Val318, Glu319, Val321, Asn322, Ser325, Ile326, Met329, Ser330, Gly332, Val333, The336, Asp337, Val338, Val341, Phe343, Leu345, Leu347, Asp348, Tyr351, Leu352, and Glu355 residues of ApoL1-G0 and Lys130, Val131, Gly132, Asp133, Gly213, Ile214, Gly215, Phe216, Gly217, Asp218, Ile219, Glu222, Gly223, Ser224, Lys226, Leu227, Arg228, The229, Arg230, Ile246, Leu247, Ser249, Leu250, Gly251, Pro252, Lys253, The254, Gln255, Phe278, Tyr280, Glu281, Gly282, Asn284, Lys301, The303, Gly304, Pro321, Phe324, Phe335, Lys339, Lys340, Pro343, Pro344, Lys346, Pro348, Lys369, Asp370, Glu371, Lys372, Ser373, The374, Leu375, Glu376, Leu530, Lys619, Arg622, Ser623, Glu626, Met627, Glu630,and Lys631 residues of CD2AP. The interface residues involved in hydrogen bond formation in the APOL1G0 and

CD2AP complex include Ser10 with Thr229 (3.59 Å), Gly187 with Gln255 (3.85 Å), Leu189 with Lys253 (3.81 Å), Met194 with Lys372 (3.62 Å), Glu201 with Lys369 (2.36 Å), Thr217 with Gly304 (3.48 Å), Thr221 with Ser373 (2.85 Å), Thr221 with Leu375 (2.72 Å), Thr221 with Leu375 (2.85 Å), Thr307 with Lys226 (3.76 Å), Ile326 with Lys346 (3.45 Å), Thr336 with Gly213 (3.73 Å). Any change in these hydrogen bonds can disrupt the interface.

S. Fig. 3C.

The interaction interface of APOL1G0 and miR193a has a total interface area of 1339.1 Å^2 and the solvation free energy gain upon formation of interface $\Delta^{i}G$ is -34.3 kcal/mol. The solvation free energy of folding for the corresponding structure ΔG is -263.1 kcal/mol. The interaction interface of APOL1G0 and miR193a includes Cys13, Trp15, Leu19, Phe20, Leu21, Gly22, Val23, Gly24, Val25, Arg26, Ala27, Lys78, Val79, Ser80, Asn83, Leu84, Leu85, Leu86, Leu87, Leu88, Trp129, His130, Gly133, Gln134, Tyr136, Arg137, Gly187, Phe257, Leu258, Asn261, and Phe265 residues of ApoL1-Go and U1, G2, G3, G4, U5, C6, U7, U8, U9, G10, C11, G12, G13, G14, C15, G16,and A17 residues of miR193a that corresponds to the miR193binding site at the 3'UTR region of the APOL1 (Fig. 3C). Also, there are 7 hydrogen bonds in the interface of APOL1G0 and miR193a complex. The interface residues involved in hydrogen bond formation in the APOL1G0 and miR193a complex include Val25 with U8 (3.77 Å), Arg26 with U9 (2.11 Å), Val79 with U9 (3.45 Å), Ser80 with G10 (3.57 Å), Leu85 with C15 (3.19 Å), Leu85 with G16 (2.80 Å), Asn261 with U9 (3.02 Å). These hydrogen bonds are critical for APOL1G0 and miRa93a binding.

Figure 4.A

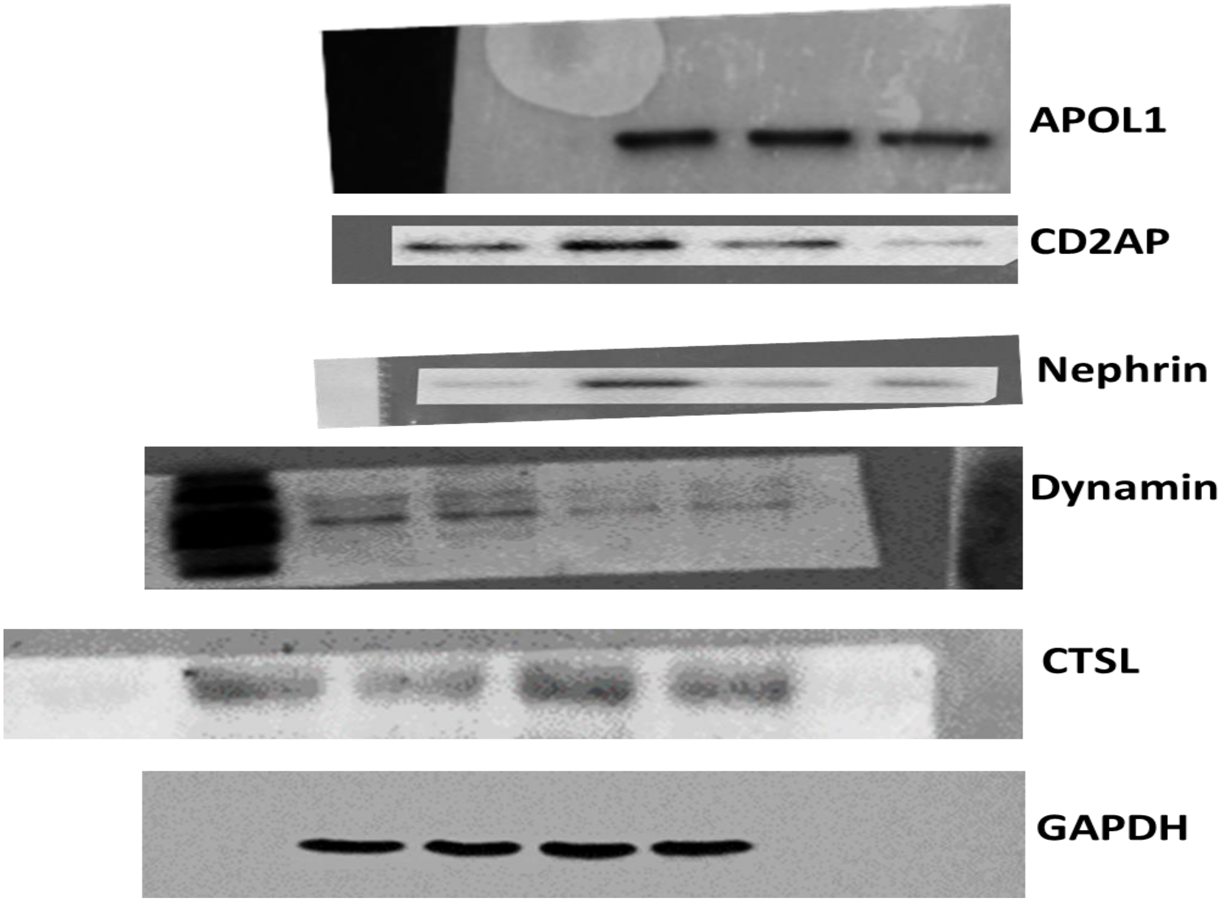

4.B

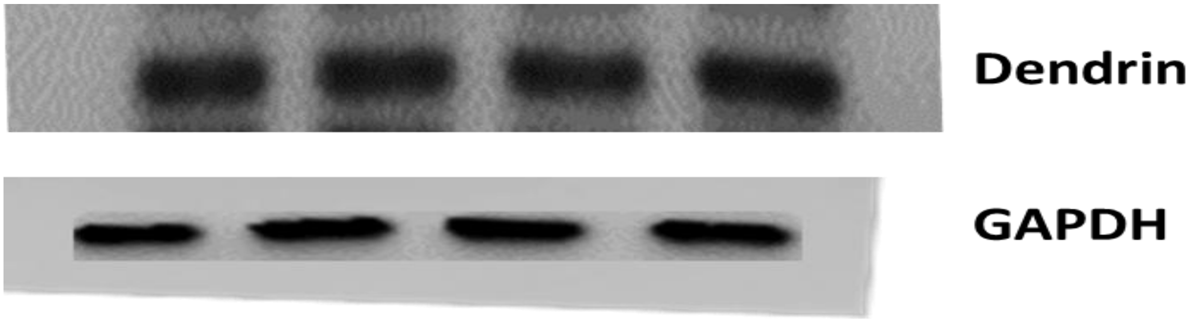

Fig . 5A

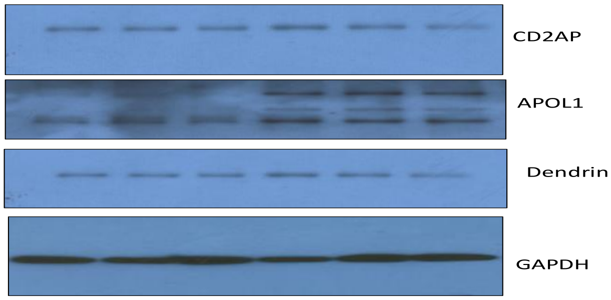

Fig . 5C

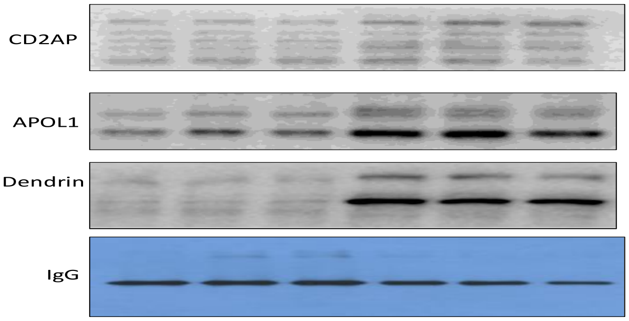

Fig 5E

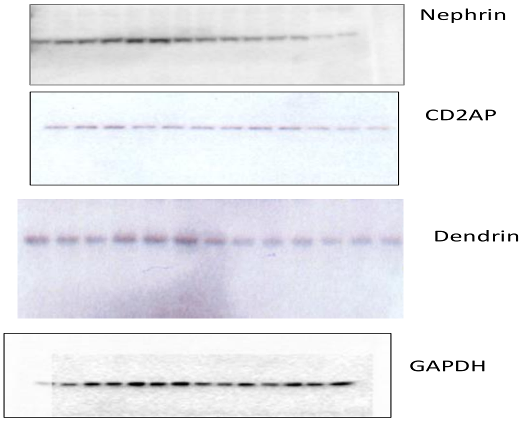

5G

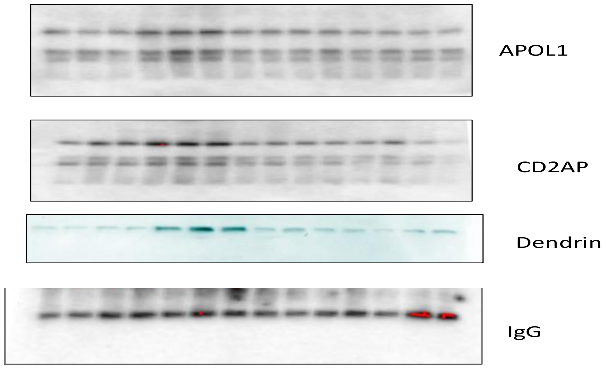

Fig. 5 I

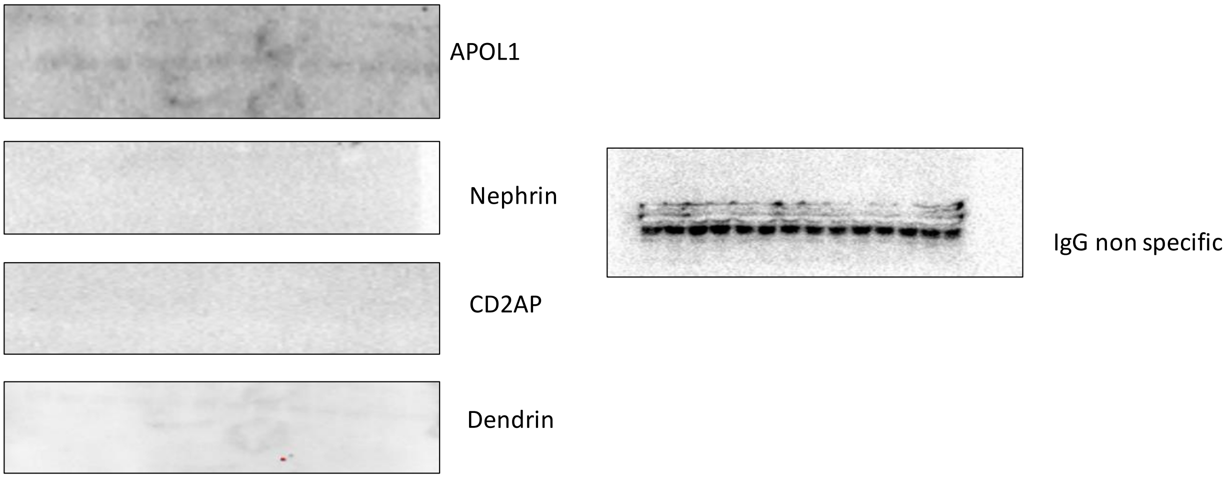

Figure 6.B

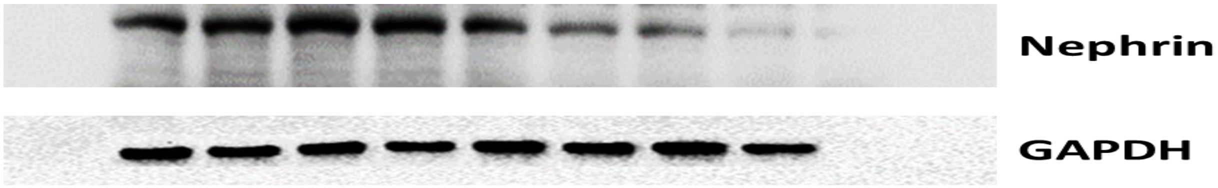

6.C

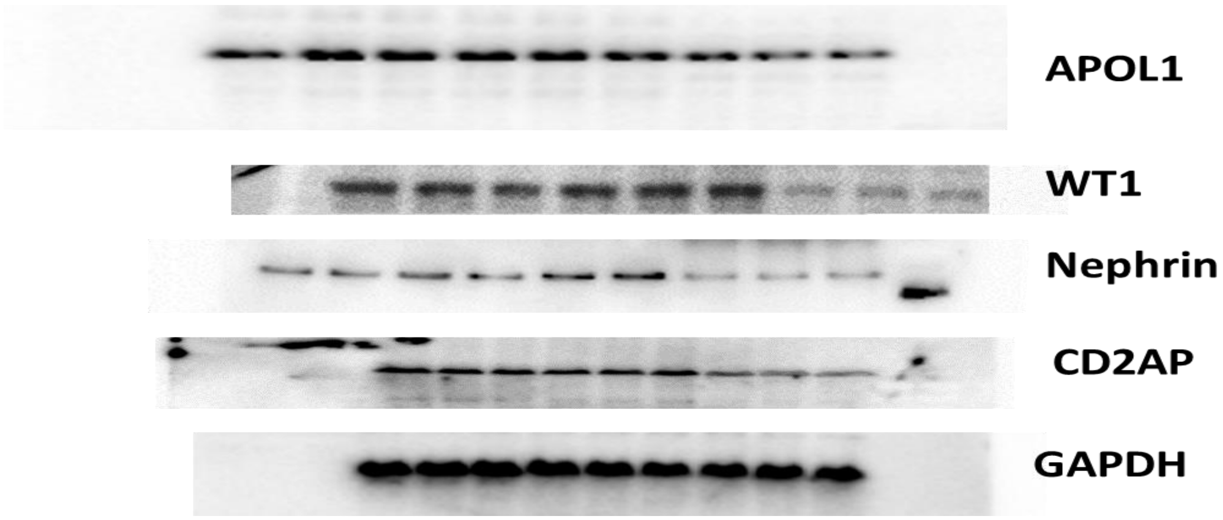

6.D

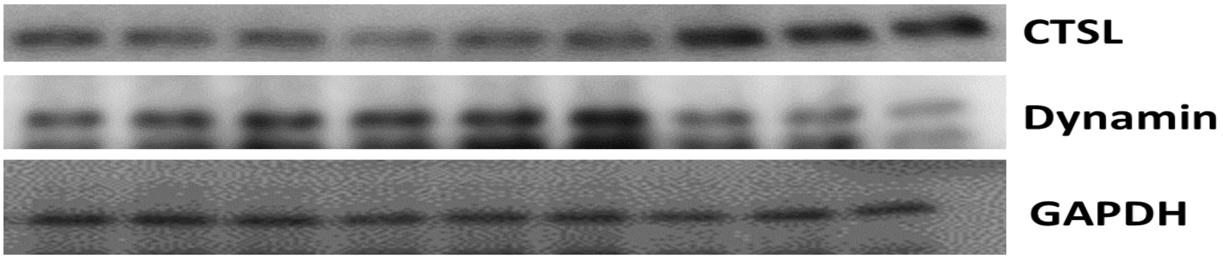

FIG 8

Figure 8.B

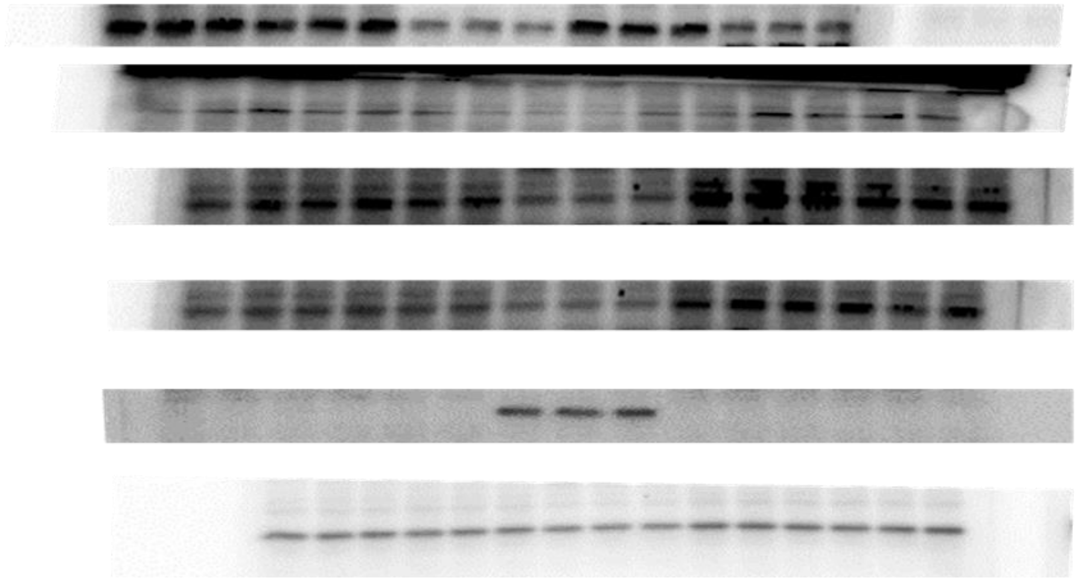

8.C

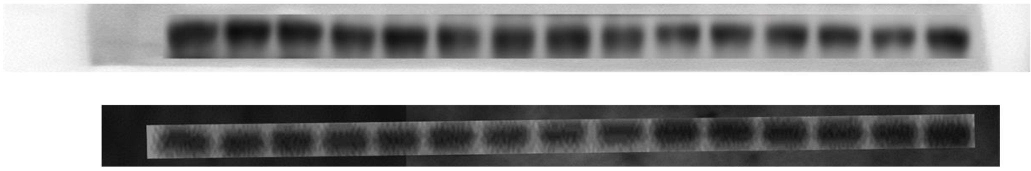

Supplement: Supplementary file 1 — Supplementary data set [file 41598_2019_39376_MOESM1_ESM.pdf]
